# Interaction between *Foxc1* and *Fgf8* during Mammalian Jaw Patterning and in the Pathogenesis of Syngnathia

**DOI:** 10.1371/journal.pgen.1003949

**Published:** 2013-12-19

**Authors:** Kimberly E. Inman, Patricia Purcell, Tsutomu Kume, Paul A. Trainor

**Affiliations:** 1 Stowers Institute for Medical Research, Kansas City, Missouri, United States of America; 2 Department of Plastic and Oral Surgery, Boston Children's Hospital, Harvard Medical School, Boston, Massachusetts, United States of America; 3 Feinberg Cardiovascular Research Institute, Feinberg School of Medicine, Northwestern University, Chicago, Illinois, United States of America; 4 Department of Anatomy & Cell Biology, University of Kansas School of Medicine, Kansas City, Kansas, United States of America; Stanford University School of Medicine, United States of America

## Abstract

Syngnathia (bony fusion of the upper and lower jaw) is a rare human congenital condition, with fewer than sixty cases reported in the literature. Syngnathia typically presents as part of a complex syndrome comprising widespread oral and maxillofacial anomalies, but it can also occur in isolation. Most cartilage, bone, and connective tissue of the head and face is derived from neural crest cells. Hence, congenital craniofacial anomalies are often attributed to defects in neural crest cell formation, survival, migration, or differentiation. The etiology and pathogenesis of syngnathia however remains unknown. Here, we report that *Foxc1* null embryos display bony syngnathia together with defects in maxillary and mandibular structures, and agenesis of the temporomandibular joint (TMJ). In the absence of *Foxc1*, neural crest cell derived osteogenic patterning is affected, as osteoblasts develop ectopically in the maxillary prominence and fuse with the dentary bone. Furthermore, we observed that the craniofacial musculature is also perturbed in *Foxc1* null mice, which highlights the complex tissue interactions required for proper jaw development. We present evidence that *Foxc1* and *Fgf8* genetically interact and that *Fgf8* dosage is associated with variation in the syngnathic phenotype. Together our data demonstrates that *Foxc1 – Fgf8* signaling regulates mammalian jaw patterning and provides a mechanistic basis for the pathogenesis of syngnathia. Furthermore, our work provides a framework for understanding jaw patterning and the etiology of other congenital craniofacial anomalies, including temporomandibular joint agenesis.

## Author Summary

Approximately one-third of all babies born with congenital defects, exhibit malformations of the head and face. Anomalies can include cleft lip, cleft palate, and abnormal development of bones and muscles. Such defects result in significant infant mortality, as well as life-long physical and social consequences for patients. Improved repair and the development of prevention strategies requires a thorough understanding of the underlying genetic, molecular, and environmental factors that contribute to normal craniofacial development and the pathogenesis of disease. In this study, we report the first genetic model of syngnathia, a rare human craniofacial defect characterized by bony fusion of the upper and lower jaw. We discovered that *Foxc1* is required for normal development of the bones and muscles of the jaw as well as the jaw joint. Our studies provide a mechanistic basis for understanding the cause of human syngnathia as well as the failure of jaw joint formation. Furthermore, our work enhances our knowledge of jaw development and may inform treatment strategies for patients with syngnathia and related craniofacial malformation conditions.

## Introduction

Jawed vertebrates, or gnathostomes, represent the majority of extant vertebrate species. In fact, more than 99 per cent of the roughly 58,000 living vertebrate species have jaws [1]. A functional, articulating jaw is required for proper nutritional intake, maintenance of oral health, and communication, and its appearance was a turning point in vertebrate evolution. Jaws allowed primitive vertebrates to become effective predators through capturing and processing large, motile prey, and probably account for much of their subsequent success in radiating and adapting to new environments.

The vertebrate jaw consists of separate upper and lower skeletal elements connected by a joint. The mature jaw structures are derived predominantly from the first pharyngeal arch (PA1), an embryonic outgrowth or facial prominence that is composed of (1) a core of mesoderm that will give rise to muscle and vasculature [2]–[4], (2) a population of neural crest cells that will differentiate into bone, cartilage, and connective tissue [5]–[8], (3) an endodermal lining, and (4) a covering of ectoderm both of which provide signals that govern proper survival, patterning, and differentiation of each of these cell populations [9]–[13]. The first pharyngeal arch can be subdivided into discrete upper (maxillary) and lower (mandibular) portions, which contribute to the upper and lower jaw respectively. In addition to these cell populations, the medial (MNP) and lateral (LNP) nasal prominences also make key tissue and signaling contributions to jaw development [14]–[18]. The jaw is constructed from several distinct and separate skeletal elements derived from these prominences including the maxilla, jugal, squamosal, and dentary bones. The temporomandibular joint (TMJ) is the functional jaw joint in mammals and is essential for jaw articulation. The TMJ is a complex synovial joint and consists of the glenoid fossa of the squamosal bone, the condylar head of the dentary, a fibrocartilaginous disc that is located between these two bones, and muscles and tendons that attach to the joint [19].

Craniofacial anomalies constitute approximately one-third of all congenital defects. Given the anatomical complexity associated with jaw development and function, it is not surprising that jaw malformations occur frequently. Syngnathia which is characterized by fusion of the upper and lower jaw, is a rare disorder for which the genetic or environmental etiology and pathogenesis remains unknown.

*Foxc1* is a member of the forkhead box winged helix transcription factor family distinguished by its highly conserved forkhead DNA binding domain (for reviews, [20], [21]). In mice, *Foxc1* has been reported to play roles in meningeal [22], [23], calvarial [24]–[28], ocular [29]–[31], somitic [32] and renal development [33]. Here we report a novel role for *Foxc1* in orofacial development. *Foxc1* null mutant mouse embryos display bony syngnathia in addition to defects in maxillary and mandibular structures together with agenesis of the TMJ. We present evidence that *Foxc1* interacts genetically with *Fgf8* to control patterning of the neural crest cell derived jaw and TMJ and furthermore that the variation in the severity of syngnathia is *Fgf8* dosage-dependent. Our results therefore demonstrate that *Foxc1* plays a critical role in jaw development and disease; provides a mechanistic basis underpinning the pathogenesis of the syngnathia; and establishes a framework for understanding the etiology of other congenital craniofacial anomalies, including temporomandibular joint agenesis.

## Results

### *Foxc1^−/−^* newborn mice display syngnathia

**Figure 1:**
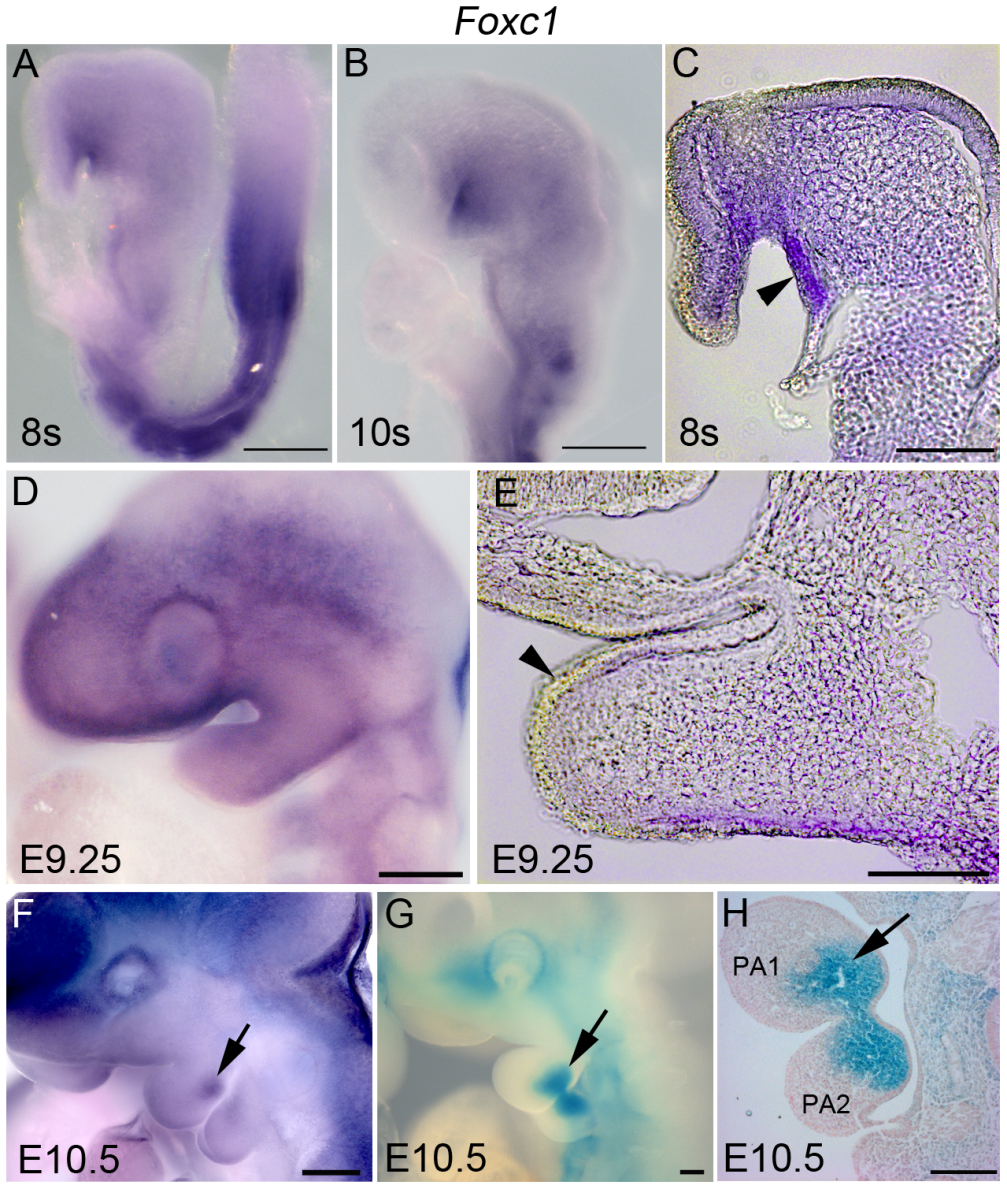
Dynamic expression of *Foxc1* in PA1. Whole mount *in situ* hybridization (A–F) and β-galactosidase (β-gal) (G, H) staining showing a timecourse of expression of *Foxc1* in the developing first pharyngeal arch. (A–C) At E8.5, *Foxc1* is broadly expressed in the cranial mesenchyme, PA1 mesenchyme and strongly expressed in the oral ectoderm. (D, E) By E9.5, expression is diffuse within PA1 mesenchyme, and is no longer detected in the PA1 oral ectoderm. (F–H) By E10.5, a discrete domain of *Foxc1* is detected in the mandibular mesenchyme, which is more easily seen in β-gal stained specimens. Scale bars: A, B, D, F–H, 200 µm; C, E, 100 µm.

*Foxc1* is initially expressed in the oral ectoderm and cranial mesenchyme in E8.5 embryos **(** **Figure 1A–C** **)**. By E9.25, *Foxc1* activity has diminished in the oral ectoderm, but continues to be expressed diffusely within the PA1 mesenchyme **(** **Figure 1D, E** **)**. At E10.5, *Foxc1* is restricted to a discrete caudal-medial domain of the mandibular portion of PA1 **(** **Figure 1F** **)**. β-galactosidase expression under the control of the endogenous *Foxc1* promoter [26] demarcates a similar spatiotemporal domain of expression in the mandibular mesenchyme **(** **Figure 1G, H** **)**. However, the stronger staining intensity may reflect the stability or persistence of *lacZ* expression over whole mount *in situ* staining. Nonetheless, this data illustrates the dynamic activity of *Foxc1* within the developing PA1 in E8.5 -10.5 embryos.

**Figure 2:**
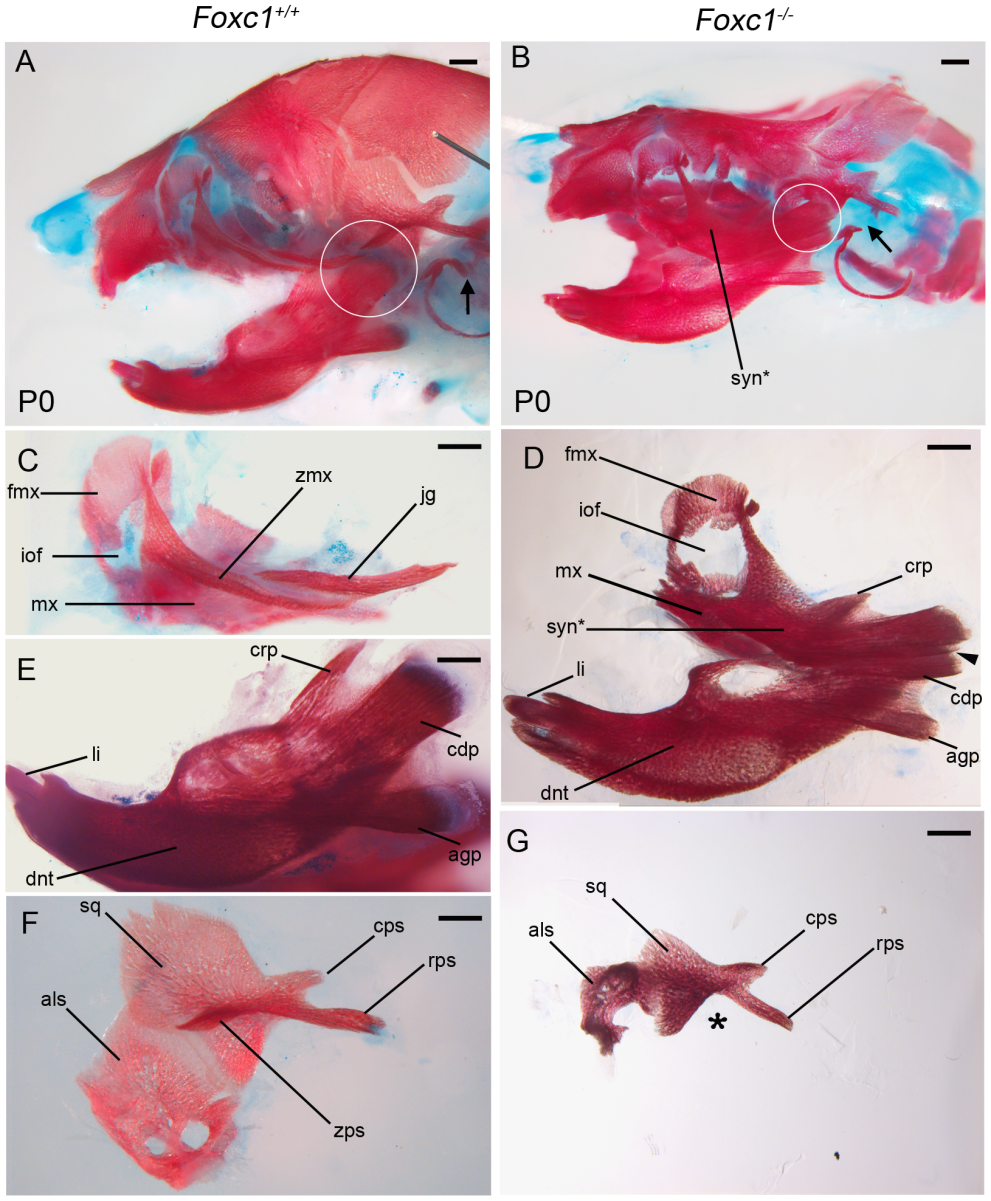
Foxc1^−/−^ neonates exhibit syngnathia and TMJ agenesis. Alizarin red (bone) and alcian blue (cartilage) stained skeletal preparations of *Foxc1^+/+^* (A, C, E, F) and *Foxc1^−/−^* (B, D, G) P0 neonates. (A,B) Intact view of cranial skeleton showing relative position of upper and lower jaw elements. Syngnathia (syn*) is evident in *Foxc1^−/−^* neonates. Circles highlight the articulating joint. Arrows in A and B highlight lack of ossification of malleus, incus, and stapes in mutant middle ear. (C) Dissected wild-type maxilla (mx) and jugal (jg). (D) The mutant maxilla is fused in the zygomatic region to the dentary (dnt) which displays hypoplastic coronoid (crp), condylar (cdp), and angular (agp) processes compared to controls (E). The *Foxc1^−/−^* condyle is bifurcated (arrowhead in D). (F, G) The mutant squamosal (sq) and alisphenoid (als) are hypoplastic, and the squamosal lacks a zygomatic process (asterisk). Scale bars: 500 µm Abbreviations: cps, caudal process of squamosal; fmx, frontal process of maxilla; iof, infraorbital foramen; li, lower incisor; rps, retrotympanic process of squamosal; zmx, zygomatic process of maxilla; zps, zygomatic process of squamosal.

We discovered that *Foxc1^−/−^* mutant mice exhibit a bilateral fusion of the upper jaw zygomatic complex to the dentary bone **(** **Figure 2A, B** **)** closely mimicking a condition in humans termed syngnathia. To evaluate the syngnathic phenotype in detail, we dissected the maxillary and mandibular structures from skeletal preparations of *Foxc1^−/−^* late gestation to newborn pups (embryonic day 18.5 – postnatal day 0; E18.5-P0 **Figure 2**). A summary of the skeletal phenotypes is presented in **Table 1**. Compared to the wild-type controls **(** **Figure 2C** **)**, the body of the maxillary bone in *Foxc1^−/−^* mutant embryos was reduced in size and the abnormally thickened and shortened zygomatic process of the maxilla was fused to the dentary bone **(** **Figure 2D** **)**. Fusion occured either proximal to the molar alveolar ridge (n = 7/13, **Figure 2D**) or encompassed the entire alveolar region (n = 6/13, **Table 1** **)**. Although this phenotype has been previously described as an enlarged zygomatic process of the maxilla [26] or as massively ossified zygomatic and dentary bones [27], our more detailed analyses indicate that *Foxc1^−/−^* mutant mice represent a unique previously undescribed model for studying the pathogenesis of syngnathia.

**Table 1:** Summary of skeletal phenotypes in jaw related structures.

	Premaxilla	Nasal Bone	Maxilla	-Body	-Palatal process	-Frontal process	-Zygomatic process	Jugal	Squamosal	Dentary	-Distal region	-Proximal region	Meckel's cartilage	-Body	-Middle ear	Facial Asymmetry	Boney Palate Fusion
***Foxc1^−/−^*** ** (n = 13)**	+	+		↓	+	+	thick, fused to alveolar (6/13) or proximal to alveolar (7/13) region of dentary	-, (12/13)	↓, no zygomatic process or glenoid fossa		+	↓, small angular and coronoid processes; bifurcated condyle; alveolar region fused to zygomatic region of maxilla		+	+, ossicles do not ossify	−	-, small region of posterior palatine bone does not fuse
***Fgf8*** **^Null/Neo^ (n = 8)**	↓, rounded and fused ventrally	−		↓	↓	↓	thick, fused at alveolar region of dentary	-	- (7/8); ↓↓(1/8)		+	↓↓, no angular, coronoid, or condylar process; fusion with zygomatic region of maxilla at aleveor region		+	↓↓, does not extend to middle ear region	++	--, all palatal processes are reduced and unfused
***Foxc1^−/−^;Fgf8^Null/+^*** ** (n = 4)**	↓	↓		↓↓	↓↓	↓↓	fused distal to alveolar region of dentary	-	-		↓	↓↓↓, no angular, coronoid, or condylar process; fusion of zygomatic region to distal dentary		+	↓, ossicles do not ossify	+	---, all palatal processes are severely reduced and unfused

Legend: +, normal; − absent; ↓, decreased; multiple arrows for a trait indicate relative severity within the category.

### Temporomandibular joint agenesis in *Foxc1^−/−^* embryos

**Figure 3:**
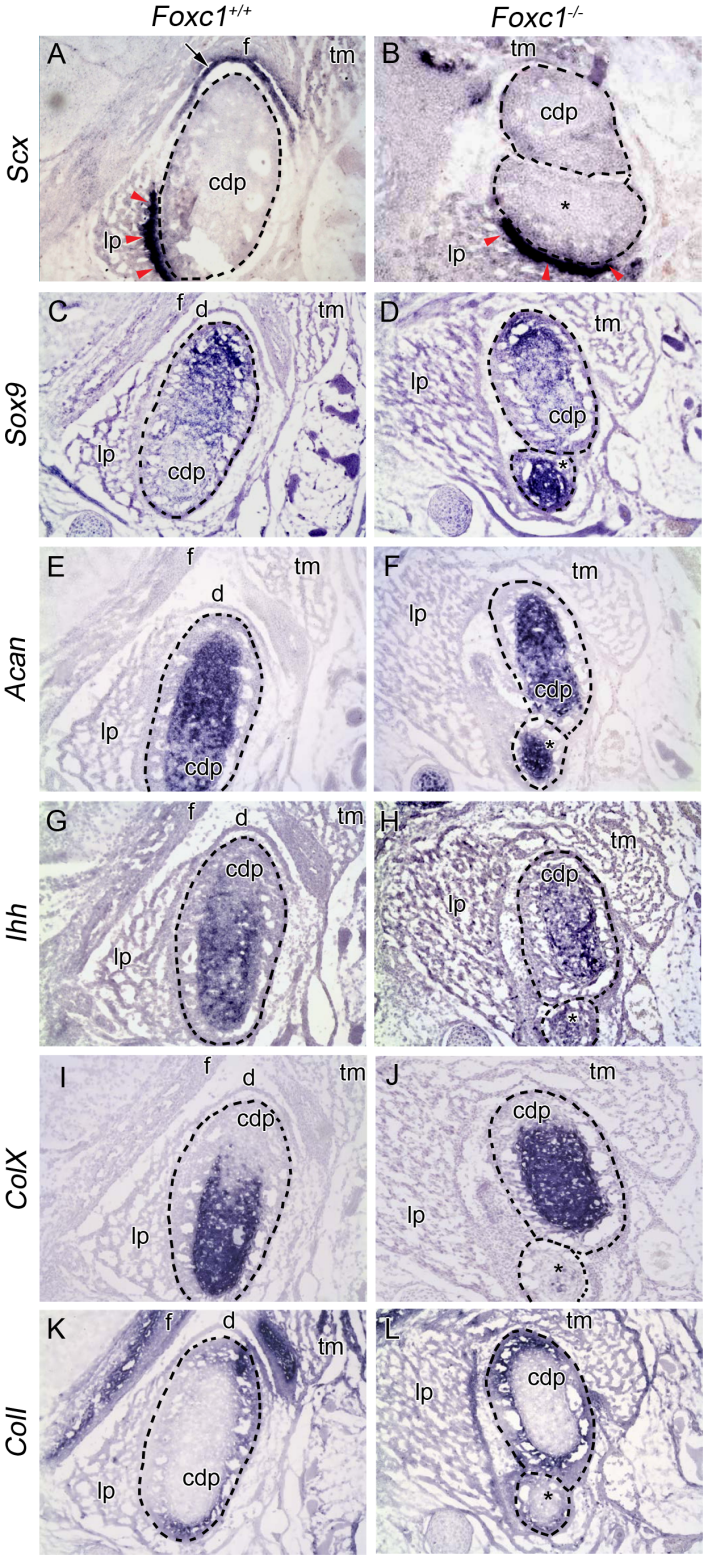
Molecular analysis of developing TMJ in *Foxc1^−/−^* embryos. *In situ* hybridization analysis on serial coronal cryosections in *Foxc1^+/+^* (A, C, E, G, I, K) and *Foxc1^−/−^* (B, D, F, H, J, L) mouse heads at E17.5. In (A–L) dashed lines outline the wild-type and bifurcated mutant condyles (cdp and cdp*). (A, B) *Scx* is expressed in the neural crest-derived disc (arrow) and tendon (red arrowheads) of controls. In mutants, Scx is maintained in the tendon (red arrowheads), but no disc is present. (C–F) *Sox9* is localized to the proliferating chondrocytes and *Acan* is localized to the cartilage of the condyle growth plate in wild-type and *Foxc1^−/−^* condylar processes. (G, H) *Ihh* is localized to the prehypertrophic chondrocytes in wild-type and mutant condyles. (I, J) *ColX* is expressed in the zone of the hypertrophic chondrocytes of the condylar growth plate. In the absence of *Foxc1*, the hypertrophic chondrocytes make up a larger proportion of the abnormally bifurcated condyle. (K, L) *Coll* expression is localized to the osteoblasts of the condyle in control and *Foxc1^−/−^* embryos; however, the *Coll* positive glenoid fossa (f) is only observed in control specimens.

The articulation of the mammalian jaw occurs at the TMJ located between the condyle of the dentary and the glenoid fossa of the squamosal bone **(circle;** **Figure 2A, C, E** **)**. The condyle in *Foxc1^−/−^* mutant embryos was hypoplastic and appeared bifurcated (n = 10/13) **(arrowhead;** **Figure 2B, D** **)**. Given the degree of fusion, we cannot however, rule out the possibility that this could also represent a duplicated angular process. Nonetheless, the squamosal body was similarly hypoplastic in *Foxc1^−/−^* mutant embryos compared to wild-type controls with no evidence of zygomatic process formation **(** **Figure 2F, G** **)**. In E17.5 wild-type embryos, a neural crest cell derived articular disc normally sits between the condyle and glenoid fossa and expresses scleraxis (*Scx*) [34] **(** **Figure 3A** **and S1A, B)**. However, no equivalent domain of *Scx* positive expression, as evidence of articular disc formation was observed around the hypoplastic condyle in *Foxc1^−/−^* embryos **(** **Figure 1C and 3B** **)**.

Although the structural features of the TMJ are well documented, the genetic, cellular, and molecular mechanisms involved in TMJ morphogenesis remain poorly understood. To explore the mechanistic basis of these jaw joint anomalies, we analyzed the expression of several genes involved in cartilage and bone formation at E17.5. All markers of proliferating and mature chondrocytes were very similar between wild-type and *Foxc1^−/−^* condyles **(** **Figure 3C–H** **)**. However, the proportion of *ColX* positive hypertrophic chondrocytes within the *Foxc1^−/−^* condyle appeared to be larger relative to controls, suggesting premature ossification **(** **Figure 3I, J** **)**. The expression of *ColI*, a marker of osteoblasts, remained unchanged in the condyle of mutant embryos **(** **Figure 3K, L** **)**, but was not seen across from the condyle indicating that no fossa formed. Collectively, our histological and molecular analyses of the TMJ in *Foxc1^−/−^* mutant embryos, illustrate the absence of glenoid fossa and articular joint disc, in addition to an abnormal condyle during TMJ development. This indicates that in addition to the syngnathia described above, a functional TMJ does not form in the absence of *Foxc1*.

### Abnormalities of the palate in *Foxc1^−/−^* embryos

**Figure 4:**
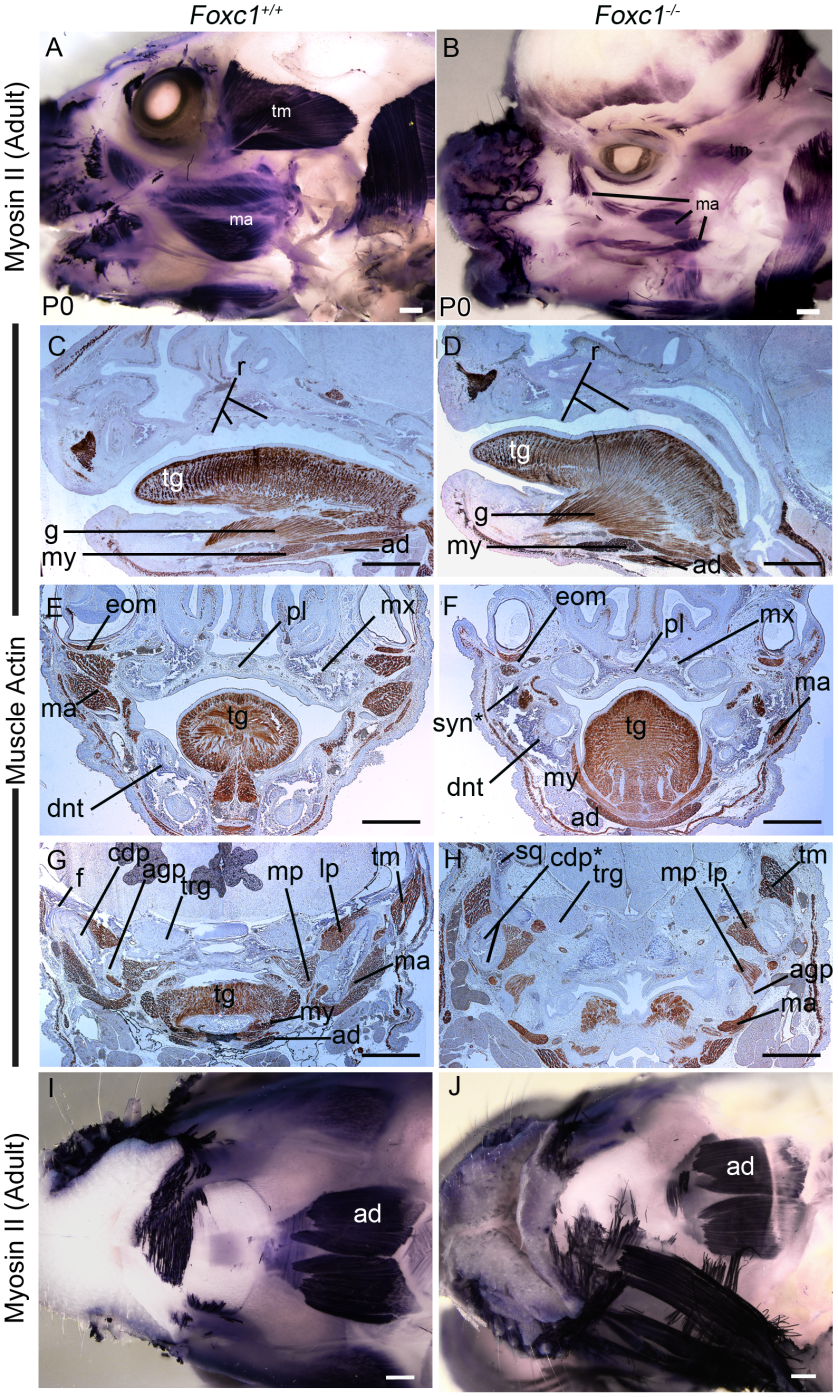
Abnormalities of PA1 derived muscle in *Foxc1^−/−^*. (A,B) Whole mount immunostaining for neonatal myosin II in P0 wild-type (A) and *Foxc1^−/−^* (B) heads. The PA1 masseter (ma) and temporalis (tm) muscles are smaller in the mutant than in wild-type. (C–G) Immunostained paraffin sections of E17.5 *Foxc1^+/+^* (C, E, G) and *Foxc1^−/−^* (D, F, H) heads showing muscle actin localization (brown). Sagittal (C, D) and frontal (E, F) sections showing organized muscle formation in both wild type and mutant tongues (tg). The palate (pl) is more arched in *Foxc1^−/−^* and shows smoother rugae (r) than in *Foxc1^+/+^*. (G, H) Frontal sections in the TMJ region. The mutant temporalis muscle (tm) is shifted medially, aberrantly associates with the bifurcated condyle (cdp*), and its fibers are oriented differently than in wild type. The medial (mp) and lateral (lp) pterygoid muscles are appropriately associated with the angular (agp) and condylar processes in *Foxc1^−/−^*. The mutant masseter (ma) is reduced to a small component in *Foxc1^−/−^* (compare ma regions in F, H to E, G). (I, J) Ventral view of whole mount myosin II immunostained P0 wild type (I) and *Foxc1^−/−^* (J) heads. The second heart field derived anterior digastric muscle (ad) is robustly detected in all specimens. Scale bars: 500 µm Abbreviations: dnt, dentary; eom, extraocular muscle; f, glenoid fossa; g, genioglossus muscle; my, mylohyoid muscle; mx, maxilla; trg, trigeminal ganglion.

Skeletal preparations at P0 revealed that in contrast to wild-type littermates, a small region of the posterior palatine bone did not fuse in *Foxc1^−/−^* neonates **(Figure S2A, C)**. The soft tissue walls of the buccal cavity were shifted medially in association with the syngnathic jaw, and consequently the palate in *Foxc1^−/−^* neonates was slightly more arched than in wild-type **(** **Figure 4C–F** **)**. The incisive papilla and rugae were readily identifiable in wild-type and *Foxc1^−/−^* palates. Whereas wild-type palates develop 8 rugae in a distinct sequence [35], only 7 rugae form in *Foxc1^−/−^* palates. Furthermore, the mutant rugae were not as sharply delineated as in wild-type (**Figure S2B, D and S4C, D)**. Despite abnormalities in the bones of the *Foxc1^−/−^* palate, no overt soft tissue clefting was observed in either the primary or secondary palate in *Foxc1^−/−^* neonates **(Figure S2B, D)**.

### Muscle patterning is abnormal in *Foxc1^−/−^* embryos

The pharyngeal arches contribute mesoderm to the tongue and the muscles of mastication – the masseter, temporalis and pterygoid. Given the jaw anomalies evident in *Foxc1^−/−^* mutant embryos, we explored whether muscle patterning was also affected. The anterior end of the tongue in *Foxc1^−/−^* mutant embryos was abnormally spade shaped and protruded from the oral cavity **(Figure S2E, F)**. Both fungiform and median circumvallate papillae were identified in the *Foxc1^−/−^* tongue, and section immunostaining for muscle actin revealed that both intrinsic and extrinsic tongue muscles were well-organized in wild-type and mutant specimens **(** **Figure 4C–F** **)**. Thus the tongue muscles form normally in *Foxc1^−/−^* mice, although the shape of the tongue may be constricted by the abnormal fusion of bony elements of the jaw.

To examine the patterns of muscle formation further, we performed whole mount immunostaining for neonatal muscle using myosin II in P0 wild-type (n = 8) and *Foxc1^−/−^* (n = 4) heads **(** **Figure 4A, B** **)**. Compared to wild-type controls the masseter and temporalis muscles were reduced in size in the mutants. In histological sections, the temporalis muscle was shifted medially into the region normally occupied by the squamosal bone **(Figure S1D–G)** and was abnormally associated with the condyle. The lateral and medial pterygoid muscles, which attach to the condyle and angular process respectively were still associated with the correct processes in *Foxc1^−/−^* specimens **(** **Figure 4G, H** **)**; however, the orientation of both the lateral and medial pteyrgoid was altered, presumably due to the abnormal position of the bones in the syngnathic *Foxc1^−/−^* jaw. The digastric muscle that arises from the second heart field mesoderm was robustly detected in whole mount neonatal muscle myosin II stained heads in both wild-type and mutants **(** **Figure 4I, J** **)**. Collectively, these data indicate that abnormal cranial muscle patterning was specific to those derived from PA1, and that these muscles were abnormally shaped, sized, and positioned possibly as a secondary result of altered cranioskeletal patterning.

### Ectopic neural crest cell derived osteoblast differentiation in *Foxc1^−/−^* embryos

The jaw, TMJ and muscle patterning abnormalities could be due to abnormal development of the neural crest cell derived PA1 mesenchyme. Therefore we examined the formation, migration, proliferation and differentiation of neural crest cells in *Foxc1^−/−^* embryos compared to their control littermates. Through *Sox10 in situ* hybridization **(Figure S3A, B)** and *Wnt1-Cre;Z/EG* lineage tracing experiments **(Figure S3C–F)**, we observed that NCC formation, migration, and contribution to PA1, the nasal prominences, and their derivatives between E9.0-12.5 embryos, were all normal in the absence of *Foxc1*. Similarly, E11.5 *Foxc1^−/−^* embryos present with a normal pattern of neurofilament immunostaining, suggesting that neural crest cell contribution to cranial ganglia and peripheral nervous system is not dependent on *Foxc1* **(Figure S3G, H)**.

**Figure 5:**
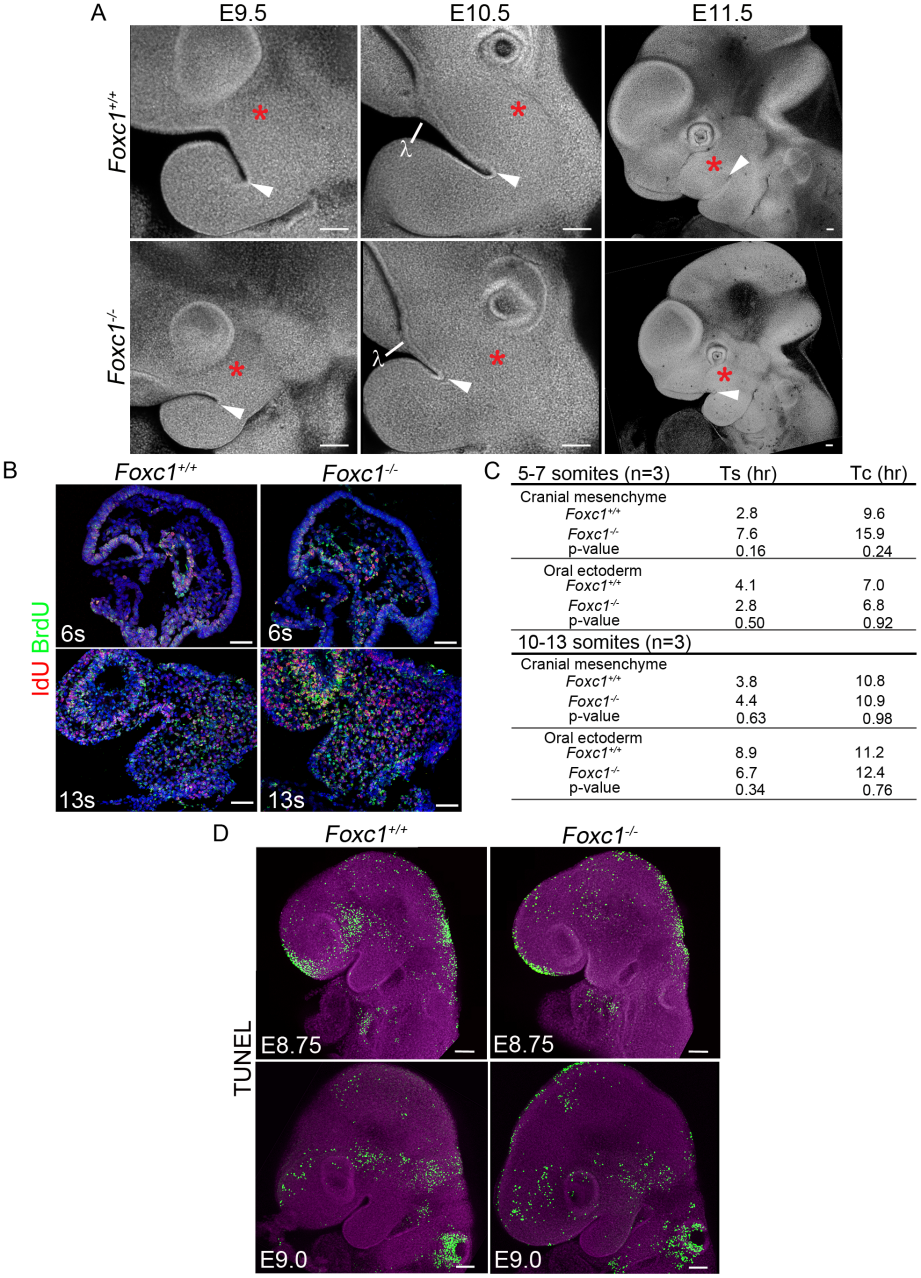
Early morphologic abnormalities of *Foxc1−/−* PA1 detected despite no significant alteration of cell cycle dynamics or apoptosis. (A) Confocal z-stack projections of DAPI stained, whole mount *Foxc1^+/+^* and *Foxc1^−/−^* embryos showing gross PA1 morphology. A red asterisk marks the maxillary prominence in all panels. A white arrowhead indicates the maxilla-mandibular junction (Mx-Md). At E9.0-9.5, the mutant maxillary prominence is smaller than wild-type, and the Mx-Md is shallower than in controls. By E10.5, the lambdoidal junction (λ) and nasal prominence epithelium has formed in *Foxc1^+/+^* and *Foxc1^−/−^* embryos. The *Foxc1^−/−^* maxillary prominence continues to develop between E10.5-11.5, but remains smaller compared to wild-type embryos. The distance from the λ to Mx-Md junction is reduced in *Foxc1^−/−^* embryos. (B) Representative sections of IdU and BrdU immunostained embryos. (C) Average S-phase and cell cycle lengths were determined based upon incorporation of IdU and BrdU. A trend toward longer S-phase and cell cycle length was noted in the cranial mesenchyme at 5–7-s. (D) Projections of confocal Z-stacks showing whole mount TUNEL staining to detect apoptotic cells in *Foxc1^+/+^* and *Foxc1^−/−^* embryos at E8.75 and E9.0. Although change in morphology of PA1 is evident, no abnormal level or localization of apoptosis is detected in the mutants. Scale bars: 100 µm.

Despite normal neural crest cell colonization, the PA1 in *Foxc1^−/−^* mutants was smaller than that of wild type littermates as early as E8.75 **(** **Figure 5A, D** **)**. From E9.5-11.5, the mutant maxillary and mandibular prominences remained reduced compared to controls and the invagination of oral ectoderm delineating the maxillary-mandibular constriction was shallower **(arrowhead;** **Figure 5A** **)**,

To determine if the smaller PA1 in *Foxc1^−/−^* embryos was a result of altered cell proliferation, we used phosphohistone H3 to quantify the mitotic index of E9.0 wild-type and mutant embryos. However, we did not observe any significant difference in the rate of cell division (p = 0.116) **(Figure S4)**. Therefore we turned to combined IdU and BrdU incorporation to determine if there were differences in cell cycle length [36] within the developing PA1 between wild-type and *Foxc1^−/−^* embryos **(** **Figure 5B, C** **)**. We detected a slightly longer S-phase and consistent with that, a longer cell cycle length in the cranial mesenchyme of *Foxc1^−/−^* embryos at the 5–7 somite stage when PA1 size difference is first apparent. Whole mount TUNEL staining revealed no ectopic or abnormal levels of programmed cell death within the pharyngeal arches of *Foxc1^−/−^* embryos between E8.75-E9.0 **(** **Figure 5D** **)**. The increase in S-phase and cell cycle length in the cranial mesenchyme can account for the smaller appearance of PA1 in *Foxc1^−/−^* compared to controls. However, our investigations into neural crest cell migration, cell proliferation, and apoptosis did not reveal any significant disruption in neural crest cell contribution to PA1 growth in *Foxc1^−/−^*, embryos. This suggested that perhaps altered neural crest cell differentiation underpinned the pathogenesis of syngnathia and TMJ agenesis.

**Figure 6:**
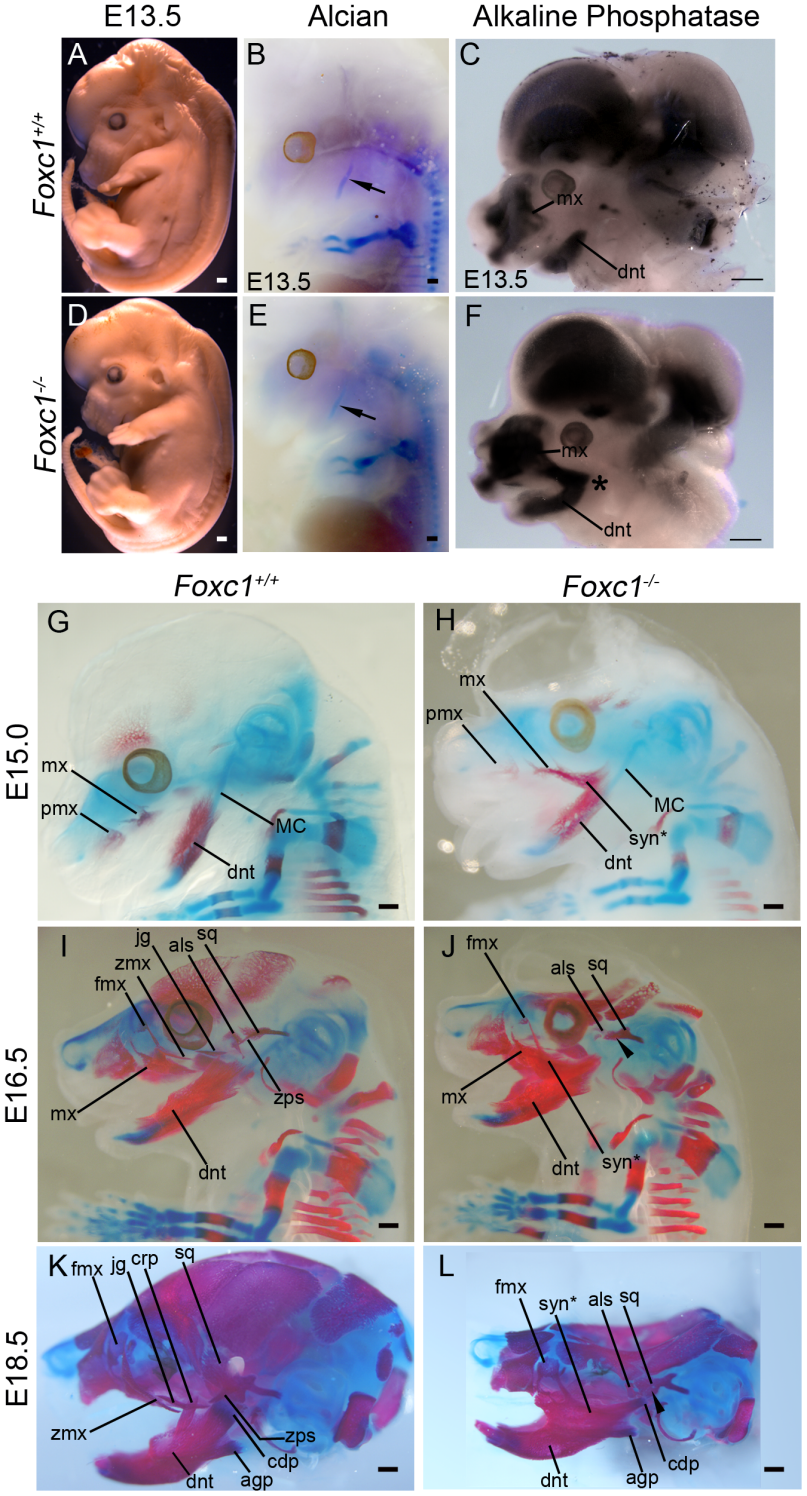
Time-course of PA1-derived skeletal abnormalities in *Foxc1^−/−^* mice. (A, D) Gross view of fixed *Foxc1^+/+^* (A) and *Foxc1^−/−^* (D) embryos at E13.5. Cerebral hemispheres are enlarged and the snout is foreshortened in the mutant. (B, E) Whole mount alcian blue staining to detect cartilage differentiation in the wild type (B) and mutant (E) embryos pictured in (A, D). A single, normal Meckel's cartilage (arrows in B, E) is seen in both control and *Foxc1^−/−^*. (C, F) Bissected E13.5 heads of *Foxc1^+/+^* (C) and *Foxc1^−/−^* (F) embryos stained for endogenous alkaline phosphatase (AP) activity to detect early osteoblast differentiation. In the absence of *Foxc1*, early osteoblasts of the dentary (dnt) and maxillary (mx) region initially differentiate in a fused, syngnathic pattern (*). (G–L) Whole mount alcian blue (cartilage) and alizarin red (bone) staining of *Foxc1^+/+^* (G, I, K) and *Foxc1^−/−^* (H, J, L) embryos. (G, H) At E15.0, ossification of the wild type maxilla (mx) with a wispy frontal process (fmx) and dentary can be seen. In the mutant, wispy projections are seen in the maxillary region similar to controls. However, this ossification is connected to a larger ossified region that is fused (syn*) directly to the dentary. (I–L) In *Foxc1^+/+^* embryos at E16.5 (I) and E18.5 (K), the zygomatic process of the maxilla (zmx), jugal (jg), zygomatic process of the squamosal (zps), and alisphenoid (als) are clearly identified as separately ossifying elements. By contrast, in the *Foxc1^−/−^* specimens (J, L), syngnathia is observed in the zygomatic region, rather than the distinct elements found in wild type. The alisphenoid and the squamosal (sq) are hypoplastic compared to controls. The zygomatic process of the squamosal does not form (arrowheads). Scale bars: 500 µm Abbreviations: agp, angular process; cdp, condylar process; crp, coronoid process; mx, premaxilla; tongue, tg.

By E13.5, *Foxc1^−/−^* embryos display foreshortened snouts compared to controls **(** **Figure 6A, D** **)**. This type of phenotype is typically associated with abnormal cranioskeletal differentiation, therefore we examined cartilage and bone differentiation in wild-type and mutant embryos via alcian blue, and alkaline phosphatase and alizarin red staining respectively. Whole mount alcian blue staining revealed a single Meckel's cartilage in both wild-type and mutant embryos which was indicative of normal cartilage differentiation **(** **Figure 6B, E, G–J** **)**. However, early neural crest cell osteogenic differentiation as detected by alkaline phosphatase staining, revealed an expanded maxillary domain that was contiguous and fused at its proximal end with the mandibular domain in E13.5 *Foxc1^−/−^* embryos in contrast to the discrete segregated domains observed in wild-type littermate controls **(** **Figure 6C, F** **)**. This preceded the manifestation of ossified bony syngnathia which was clearly evident in alizarin red stained E15.0 *Foxc1^−/−^* embryos and which progressively worsened through E16.5-18.5 as the craniofacial skeleton matured **(** **Figure 6G–L** **)**. In addition to the presence of syngnathia the bony elements of the mandible and maxilla of *Foxc1^−/−^* embryos exhibited abnormal morphology and the alisphenoid and squamosal were hypoplastic and malformed compared to wild-type controls. Thus, the pathogenesis of syngnathia in association with maxillomandibular malformation correlates with altered patterns of neural crest cell derived osteogenic differentiation.

### Proximal and distal PA1 markers are normally expressed in absence of Foxc1

After the completion of their migration into the facial prominences, neural crest cells find themselves surrounded by epithelia, and many studies have demonstrated the critical role played by epithelial-mesenchymal interactions in cranioskeletal patterning. Thus patterning of the craniofacial skeleton can be best understood when considered as modules of neural crest progenitors within territories such as the pharyngeal arches, where the signals from the surrounding epithelia interact to generate neural crest cell derived skeletal elements of appropriate size and shape. To explore whether the ectopic osteoblast differentiation and syngnathia observed in *Foxc1^−/−^* embryos was a consequence of altered pharyngeal signaling we examined the expression of markers known to play key roles in patterning the proximal-distal axis of PA1 [14], [37]–[39]. In E9.5-10.5 wild-type and *Foxc1^−/−^* mutant embryos, the lateral and medial nasal prominences develop such that morphologically the lambdoidal junction is readily identifiable **(** **Figure 5A** **)**. *Bmp4*, which is a key regulator of distal jaw patterning **(Figure S5A–D)** and its downstream target *Msx2* **(Figure S5E–H)** were expressed normally in the distal mandibular ectoderm, the olfactory epithelium, and at the lambdoidal junction in *Foxc1^−/−^* (n = 4) embryos at E10.5.

Endothelin signaling is also required to pattern the distal elements of the jaw but primarily establishes mandibular identity [40]–[43]. *Hand2*, which is a downstream target of endothelin, was expressed normally in the distal PA1 mesenchyme of *Foxc1^−/−^* (n = 4) embryos **(Figure S5I–L)**. Additionally, *Gata3* which is required for endothelin-independent expression of *Hand2* in the distal mandiblular arch [44], was also properly expressed in both the distal mandibular ectoderm and the lambdoidal junction in E10.5 *Foxc1^−/−^* embryos (n = 3) **(Figure S5M–P)**. Thus the normal expression of distal jaw signaling factors such as *Bmp4*, *Msx2*, *Hand2 and Gata3* is consistent with the normal morphology observed in the distal regions of the dentary and maxilla in *Foxc1^−/−^* embryos **(** **Figure 2A–D** **)**.

### *Fgf8* expression in PA1 is altered in the absence of Foxc1

**Figure 7:**
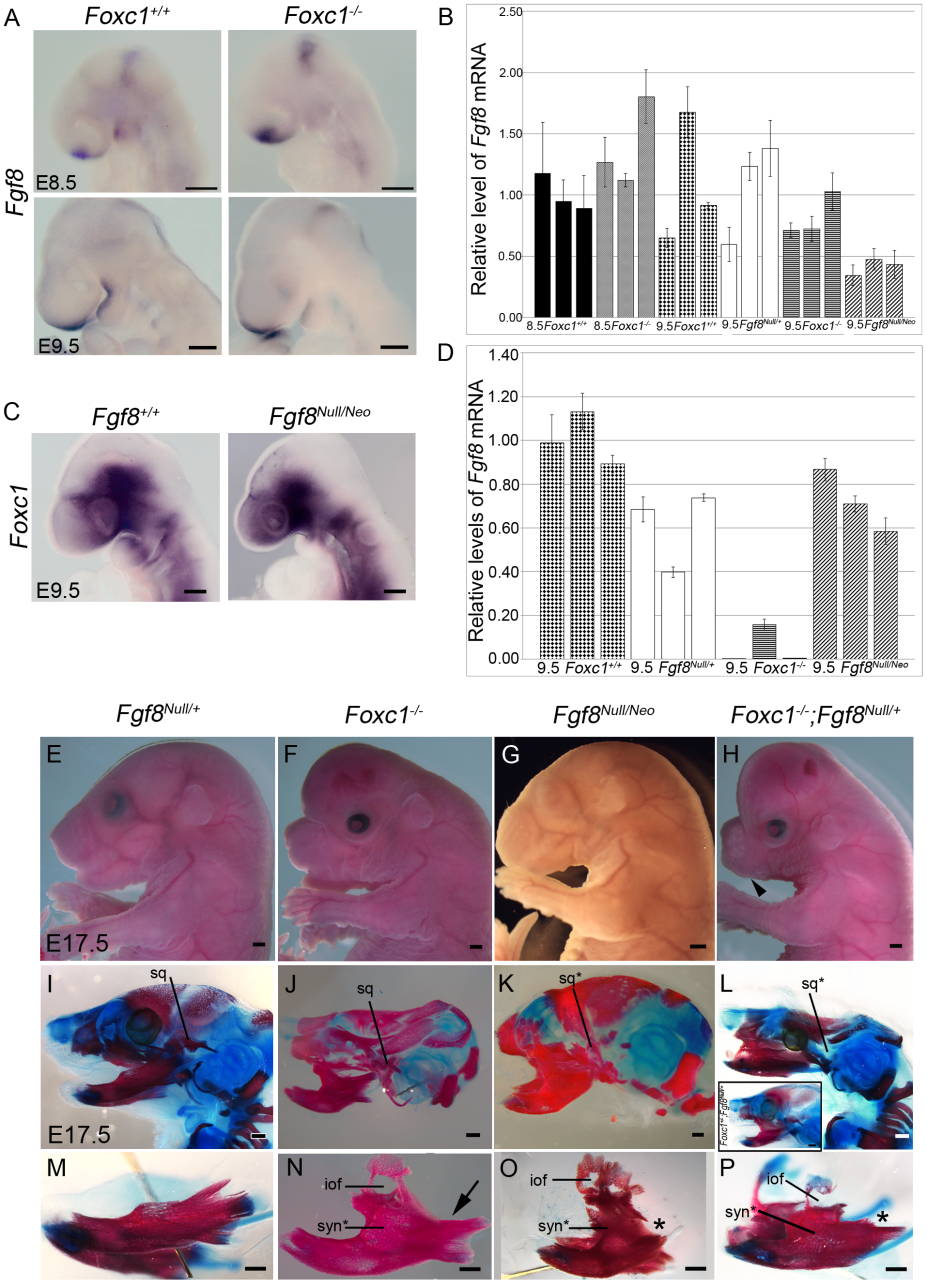
*Foxc1* is required to maintain *Fgf8* signaling and genetically interacts with *Fgf8*. (A) *Fgf8* expression is maintained in the frontonasal prominence and midbrain-hindbrain boundary regions of *Foxc1^−/−^* embryos, but it is reduced in the PA1 oral ectoderm (red asterisks). (B) Quantification of *Fgf8* mRNA in *Foxc1*, *Fgf8^Null/+^*, and *Fgf8^Null/Neo^* embryos. (C) *Foxc1* is normally localized in *Fgf8^Null/Neo^* embryos. (D) Quantification of *Foxc1* mRNA in *Foxc1*, *Fgf8^Null/+^*, and *Fgf8^Null/Neo^* embryos. Gross appearance (E–H) and skeletal preparations (I–P) of E17.5 embryos comparing *Fgf8^Null/+^* (E, I, M), *Foxc1^−/−^* (F, J, N), *Fgf8^Null/Neo^* (G, K, O), *Foxc1^+/−^*; *Fgf8^Null/+^* (L, inset), and *Foxc1^−/−^;Fgf8^Null/+^* (H, L, P) phenotypes. In both gross view (E) and skeletal preparations (I, M), *Fgf8^Null/+^* are indistinguishable from wild-type embryos. (F) *Foxc1^−/−^* embryos have shortened frontonasal regions, open eyelids, abnormal and shifted external ears, and enlarged, hydrocephalic cerebral hemispheres. (G) *Fgf8^Null/Neo^* embryos have a more rounded frontonasal region, small lower jaw, and abnormal, shifted external ears. (H) Compound *Foxc1^−/−^;Fgf8^Null/+^* embryos resemble *Foxc1^−/−^* specimens, but have more severe frontonasal shortening and no externally visible oral opening/lower jaw (black arrowhead). (J, N) Hypoplastic squamosal (sq), syngnathia (syn*), and abnormal condyle formation in the absence of *Foxc1*. This specimen shows fusion in alveolar region of dentary and absence of the coronoid process (arrow in N). (K, O) Severe hypoplasia and malformation of the squamosal (sq*) was observed in *Fgf^Null/Neo^* specimens. The frontal process of the maxilla with a characteristic infraorbital foramen (iof) formed, and the maxilla fused to the dentary in the alveolar region, more distally than seen in *Foxc1^−/−^*. The proximal processes of the dentary are absent (asterisk in O), but distal incisors form. (L, P) In *Foxc1^−/−^;Fgf8^Null/+^* embryos, the syngnathic phenotype is further exacerbated. No squamosal formed and a small frontal process of the maxilla is attached to the hypoplastic maxilla. This region is fused to the dentary just proximal to the lower incisors resulting in flattening of the normally curved dentary. The proximal dentary is severely truncated and lacks all processes (asterisk in P). (inset in L) *Foxc1^+/−^;Fgf8^Null/+^* compound heterozygote (2/12) in which calvaria had developed normally. This specimen also displayed a syngnathic jaw with TMJ abnormalities grossly identical to that of the *Foxc1* null. Scale bars: (A,C) 200 µm; (G–H) 1000 µm; (M–P) 500 µm.

Since patterning cues at the distal ends of PA1 were not altered in *Foxc1^−/−^* embryos, we hypothesized that the ectopic osteoblast differentiation and syngnathia reflected altered specification or signaling within the proximal region of PA1. Experimental evidence has implicated *Fgf8* as being essential for proximal-distal axis specification and signaling [11], [45]–[49], and it is localized to the oral ectoderm of PA1 near the maxillary-mandibular constriction. Although the oral ectoderm was clearly present, its invagination at the maxillary-mandibular constriction was shallower in E9.0-9.5 *Foxc1^−/−^* embryos compared to wild-type **(** **Figure 5A** **)**. Therefore we examined *Fgf8* expression in wild-type and *Foxc1^−/−^* embryos **(** **Figure 7A**; n = 4). In *Foxc1^−/−^* embryos, *Fgf8* expression was expressed at the midbrain-hindbrain boundary and in the frontonasal prominence similar to wild-type controls. However, *Fgf8* was reduced in the mandibular oral ectoderm and was absent from the maxillary regions of the oral ectoderm in E8.5-9.5 *Foxc1^−/−^* embryos. .

Therefore we hypothesized that diminished Fgf8 activity correlated with the pathogenesis of syngnathia. To determine if alterations of *Fgf8* gene dosage could produce a similar syngnathic phenotype, we examined the PA1 derived upper and lower jaw bones in *Fgf8^Null/+^* and *Fgf8^Null/Neo^* embryos **(** **Figure 7E, I, M, G, K, O** **)**. Indeed E18.5 *Fgf8^Null/Neo^* embryos displayed syngnathia (7/8) in which the maxilla containing a distinctive frontal process was fused to the malformed dentary, which lacked a condylar process. The site of maxilla-dentary fusion was positioned more distally in *Fgf8^Null/Neo^* embryos than in *Foxc1^−/−^* embryos **(** **Figure 7J, K, N, O** **)**. Left-right side differences were often observed in the length of each dentary bone and the location of syngnathia along the proximal-distal axis of *Fgf8^Null/Neo^* mutants. As each side of the mutant lower jaw was fused at the mandibular symphysis, this produced a distinct asymmetrical shift of the lower jaw (**Figure S6G, H**). Additional skeletal phenotype details are provided in **Table 1** **,** **Figure 5** **and Figure S6)**.

These results indicate that the relative levels of *Fgf8* in the developing PA1 are key to the pathogenesis of syngnathia. Therefore we quantified the comparative levels of *Fgf8* in each of the mutant *Foxc1* and *Fgf8* lines by qPCR **(** **Figure 7B** **)** and observed *Fgf8* activity in the oral ectoderm of *Foxc1^−/−^* embryos to be approximately only 80% of wild-type levels. This was a considerable decrease, well below the levels of *Fgf8* maintained by *Fgf8^Null/+^* embryos (100% of wild-type), but not quite as severely reduced as in *Fgf8^Null/Neo^* embryos (41% of wild type). Collectively these expression data indicate that *Foxc1* may be required to maintain proper levels of *Fgf8* in the PA1 oral ectoderm.

### Genetic interaction of *Foxc1* and *Fgf8*

Since genetically reduced levels of *Fgf8* resulted in syngnathia and *Fgf8* levels were reduced in *Foxc1* mutants, we hypothesized that *Foxc1* and *Fgf8* may genetically interact to influence jaw development. Therefore we generated compound heterozygous *Foxc1^+/−^;Fgf8^Null/+^* and *Foxc1^−/−^;Fgf8^Null/+^* embryos. Four *Foxc1^−/−^;Fgf8^Null/+^* embryos were recovered between E18.5 – P0, which closely resembled *Foxc1^−/−^* embryos, but the phenotype was much more severe that either *Foxc1^−/−^ or Fgf8^Null/Neo^* embryos, lacking an oral opening and a visible lower jaw **(arrowhead** **Figure 7H** **)**. In skeletal preparations, *Foxc1^−/−^;Fgf8^Null/+^* had a more severe phenotype than that of either *Foxc1^−/−^* or *Fgf8^Null/Neo^* embryos **(** **Figure 7I–P** **)**. The premaxilla was smaller than controls and the maxilla consisted of a small frontal process and hypoplastic body of the maxilla. The maxilla was fused to a truncated dentary that lacked all proximal processes. The site of fusion was located quite distally along the length of the dentary, nearing the incisor region, however, incisor formation occurred in each of these mutants. Meckel's cartilage was present, but the proximal portion that gives rise to the malleus was thin and was associated with a small remnant of the incal cartilage **(Figure S6A–C)**. Similar to *Foxc1^−/−^* the *Foxc1^−/−^;Fgf8^Null/+^* mutant middle ear ossicles were still cartilaginous with no evidence of stapes formation at P0, whereas the wild-type malleus and incus were undergoing ossification.

The skeletal phenotypes of the *Foxc1^−/−^;Fgf8^Null/+^* reflected an exacerbated loss of maxillary and TMJ related structures and a distal shift in the location of fusion along the proximal-distal axis of the dentary. We also observed facial asymmetry in these embryos similar to that observed in *Fgf8^Null/Neo^* embryos **(Figure S6G–J)**. Interestingly, we also occasionally observed syngnathia in compound *Foxc1^+/−^;Fgf8^Null/+^* heterozygotes at E18.5 (n = 2/12) **(** **Figure 7L** **, inset)**. Abnormalities of the maxilla, dentary, squamosal, and alisphenoid were identical to those seen in *Foxc1^−/−^* embryos. This lends further evidence for the genetic interaction between *Foxc1* and *Fgf8*. Consistent with this idea, we assessed whether *Foxc1* expression was altered by decreased levels of *Fgf8*. While *Foxc1* was properly localized to the PA1 mesenchyme, qPCR analysis indicated a slight decrease in *Foxc1* mRNA levels in both E9.5 *Fgf8^Null/+^* (59% of wild type) and *Fgf8^Null/Neo^* (70% of wild type) embryos **(** **Figure 7C, D** **)**. Thus *Fgf8* may be required to maintain proper levels of *Foxc1* in the PA1 mesenchyme and conversely *Foxc1* may be required to maintain proper levels of *Fgf8* in the PA1 oral ectoderm forming a feedback loop critical for jaw development and in the pathogenesis of syngnathia and TMJ agenesis.

### The proximal Dlx code is disrupted in PA1 of *Foxc1^−/−^* embryos

**Figure 8:**
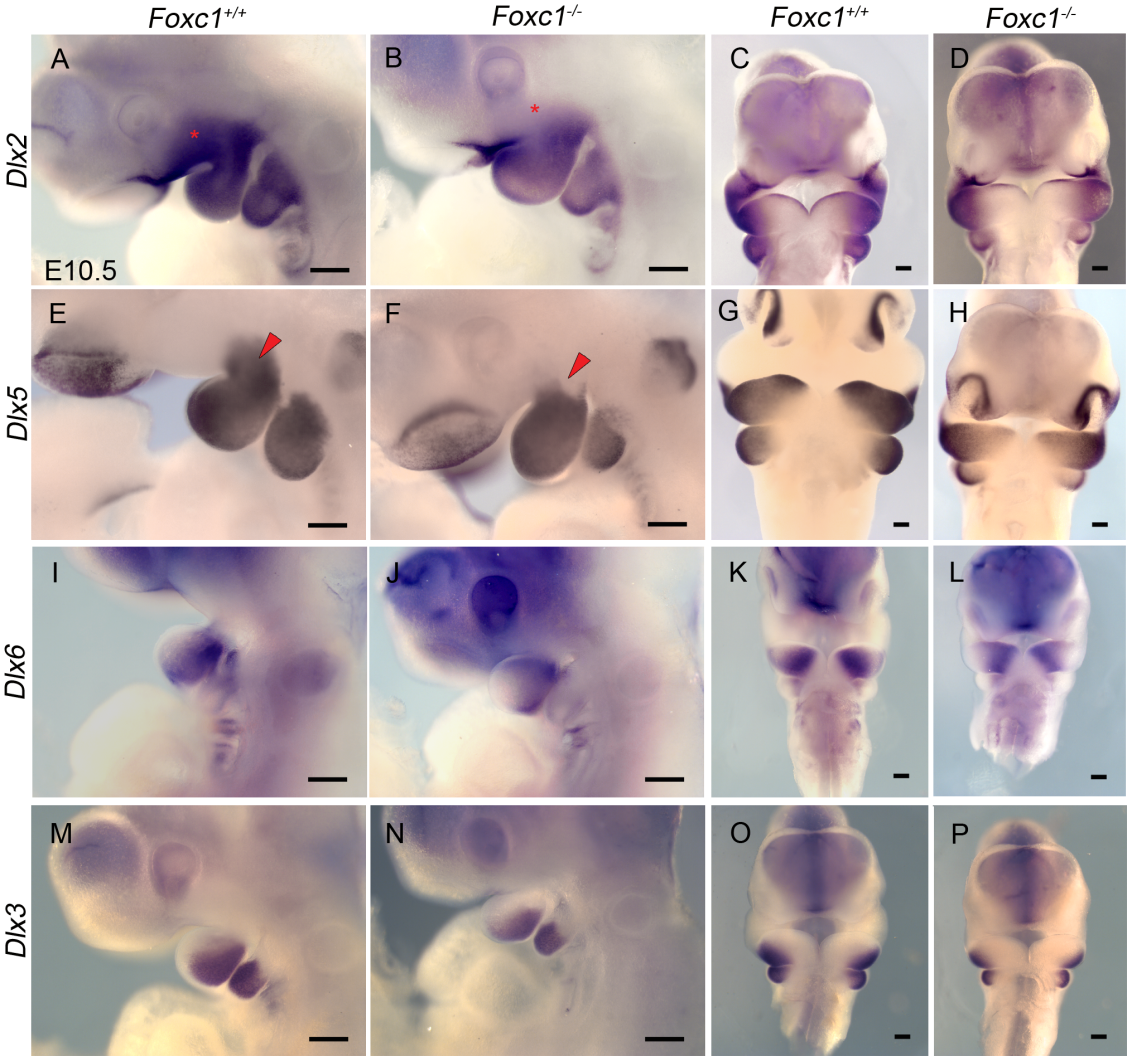
Disruption of proximal *Dlx* code in PA1 of *Foxc1^−/−^* embryos. Whole mount *in situ* hybridization in E10.5 *Foxc1^+/+^* (A, C, E, G, I, K, M, O) and *Foxc1^−/−^* (B, D, F, H, J, L, N, P) embryos. Lateral (A, B, E, F, I, J, M, N) and frontal (C, D, G, H, K, L, O, P) views are shown for each probe. (A–D) *Dlx2* expression is maintained in the λ-junction and distal mandibular ectoderm and the mandibular mesenchyme of *Foxc1^−/−^* embryos. However, expression of *Dlx2* is much reduced in the maxillary PA1 mesenchyme in the mutants (red asterisks). (E–H) *Dlx5* expression is maintained in nasal prominence epithelia, distal mandibular ectoderm, and most of the mandibular mesenchyme in *Foxc1^−/−^* embryos. A discrete domain of mesenchymal *Dlx5* expression is lost at the Mx-Md junction (red arrowheads) in the absence of *Foxc1*. In *Foxc1^−/−^* both *Dlx6* (I–L) and *Dlx3* (M–P) are expressed similarly to controls. Scale bars: 200 µm.

Interestingly Fgf8 is instrumental in activating *Dlx* genes in neural crest cells thereby triggering the morphogenetic program which specifies regionalized jaw elements [50], [51]. Regionalized patterning within PA1 is elaborated through a nested proximal-distal code of *Dlx* homeobox transcription factors in the mesenchyme [52]–[57], and the interpretation, pattern, and morphology of the jaw depend on the combinatorial activity of *Dlx* genes [56]. Therefore we examined the expression of *Dlx* genes in neural crest derived pharyngeal arch mesenchyme **(** **Figure 8** **)**. *Dlx2* expression in the maxillary portion of PA1 was reduced or absent in E10.5 *Foxc1^−/−^* mutant embryos compared to wild-type controls (n = 4) **(** **Figure 8A–D** **)**. Similarly, there was a distinct loss of *Dlx5* expression in the mesenchyme at the maxillary-mandibular constriction *Foxc1^−/−^* mutant embryos (n = 4) **(** **Figure 8E–H** **)**. In contrast, the expression domains of both *Dlx3* and *Dlx6* appeared to be normal in *Foxc1^−/−^* embryos (n = 3) **(** **Figure 8I–P** **)**. Therefore, the syngnathia and TMJ agenesis observed in *Foxc1^−/−^* embryos likely manifests as a result of perturbed Fgf8 signaling in the proximal region of PA1, together with alterations in distinct subdomains of *Dlx2* and *Dlx5* which collectively lead to regionalized patterning defects in maxillary and mandibular mesenchyme from which the upper and lower jaw elements are derived.

## Discussion

Syngnathia (fusion of the upper and lower jaw) is a rare human condition with only 56 cases reported to date in the literature (**Table S1**, and references therein). Syngnathia may involve connection between soft tissues (synechiae) or union between bony elements (synostosis). Bony fusion is rare, requires complex surgical repair, and is often present as part of a broader syndrome of congenital malformation. These cases reveal a high degree of variability in the location and extent of jaw fusion and indicate that bony syngnathia may be isolated or syndromic. In about 18% of cases, syngnathia is associated with known syndromes such as aglossia-adactylia syndrome or hemifacial microsomia. In all the reports of bony syngnathia, there is bony fusion between elements of the upper and lower jaw and no known epigenetic, genetic, or molecular etiology.

Previous reports have speculated that congenital bony syngnathia is caused by various factors, including abnormal development of the stapedial artery [58]; late gestation trauma or pressure defects *in utero* [59], [60]; and abnormal neural crest cell proliferation and migration [61]. Our review of the case reports suggested that syngnathia arises from defects in pharyngeal arch development and that there was likely a molecular or genetic basis for this disorder. In this study we present the first genetic model, a targeted deletion of *Foxc1*, with which to further understand the etiology and pathogenesis of human syngnathia.

### *Foxc1* knockout mice provide a model for understanding human syngnathia

Similar to human bony syngnathia, fusion of the zygomatic complex to the dentary bone in *Foxc1^−/−^* mutants occurred distal to the temporomandibular joint region. Furthermore, components of the TMJ were also abnormal in the *Foxc1^−/−^* model. Defects were observed in the morphology of the condyle and squamosal, in addition to agenesis of the joint disc and glenoid fossa. Our studies also indicate that jaw associated muscles, including the masseter, temporalis, and pterygoids, were deficient and abnormally positioned in the absence of *Foxc1*. This reduction in size of the muscles of mastication is very similar to the decrease in muscle volume of the masseter, temporalis, and pterygoid reported in human hemifacial microsomia patients [62]–[65].

### Foxc1 plays a role in patterning of maxillary and joint related structures of jaw

Abnormal morphology was observed in *Foxc1^−/−^* as early as E8.75 in the form of maxillary and mandibular hypoplasia, with the maxillary prominence more grossly affected. This occcured in the absence of changes in neural crest cell contribution to the arch, or decreases in the mitotic index, or ectopic apoptosis. We did observe a lengthening of the cell cycle in *Foxc1^−/−^* cranial mesenchyme, which may contribute to the reduced size of PA1. It is also possible that morphological shape changes contribute to the reduction of the PA1 in *Foxc1^−/−^* embryos. Future morphometric analyses may shed further light on the underlying cause of the gross abnormalities of PA1 in *Foxc1^−/−^* embryos

Our data indicate that the epithelial signals associated with the distal regions of PA1 remain intact in the absence of *Foxc1*. We investigated known growth signaling and transcription factors associated with regionalized patterning of PA1 and subsequent jaw development. Reduction in *Dlx2* was restricted to the maxillary prominence mesenchyme and loss of *Dlx5* was limited to a discrete region of mesenchyme at the maxillo-mandibular constriction. The loss of *Foxc1* has the most dysmorphic effect on the maxilla, jugal, and squamosal indicating a requirement for *Foxc1* in maxillary patterning and differentiation, particularly structures of the zygomatic complex. The zygomatic complex is also disrupted when *Dlx2* is knocked-out in mouse [53]. In addition, targeted disruption of *Dlx5* [54] results in abnormal truncation and arrangement of the condylar and angular processes as well as a deviation or split in Meckel's cartilages associated with abnormal ossification. These structures are similarly abnormal in *Foxc1^−/−^* mutants. Combinatorial heterozygotic loss of *Dlx2* and *Dlx5* [56], results in abnormalities of maxillary, palate, and dentary structures as well as severe disruption of both the primary (incal-malleal) and secondary (squamosal-condylar) articulations. Interestingly, loss of *Dlx2/5* also results in loss of PA1 derived branchiomeric muscle [66] while *Foxc1^−/−^* mutants exhibit significant reduction and abnormality of these muscles. Our data showing discrete changes in the proximal *Dlx* code and resultant skeletal phenotype in *Foxc1^−/−^* mutants is consistent with the previous studies of nested *Dlx* expression in jaw patterning, and recent studies in mouse indicating that *Dlx* expression within the arch may be more dynamic than previously appreciated [67]. It appears that the specific levels and domains of *Dlx2* and *Dlx5* expression that require *Foxc1* are critical for patterning elements of the zygomatic complex, maxilla, squamosal and proximal dentary upon which mammalian jaw articulation depends.

### *Fgf8* dosage affects severity and phenotypic variability in syngnathia

Alteration to the *Dlx* code in the *Foxc1^−/−^* PA1 appears to occur downstream of *Fgf8*. Interestingly the ectodermal domain of *Fgf8* overlies the mesenchymal domain of *Dlx2* while *Bmp4* overlies the epithelial domain [68]. Moreover, the mesenchymal expression of *Dlx2* is positively regulated by Fgf8 signaling while the ectodermal activity of *Dlx2* is maintained by Bmp4 signaling. Fgf8 therefore is instrumental in activating *Dlx* genes in neural crest cells and triggering the morphogenetic program, which specifies different jaw elements [50], [51]. Consistent with this, we demonstrated that simply reducing the overall dosage of *Fgf8* could produce bony syngnathia. *Fgf8^Null/Neo^* embryos exhibit jaw fusion similar to that observed in *Foxc1^−/−^* embryos. Furthermore, genetically reducing one copy of *Fgf8* in combination with the *Foxc1* null further exacerbated the extent of jaw fusion and the perturbation of maxillary and dentary elements. Together these data indicate that *Foxc1* genetically interacts with *Fgf8* to influence jaw patterning, and that overall dosage of *Fgf8* affects the severity of the syngnathic phenotype.

The *Foxc1^−/−^* syngnathic phenotype is similar to, but less severe than the *Fgf8^Null/Neo^* jaw phenotype (see **Table** 1; **Figure 7** **; Figure S6)**. However, the combinatorial loss of *Foxc1* and *Fgf8* displayed the most severe phenotype in the spectrum reported here, which may indicate that *Foxc1* and *Fgf8* function synergistically in their roles in jaw patterning. Interestingly, as *Fgf8* dosage decreased in these mouse mutants, we observed facial asymmetry similar to that reported in human syngnathia [69], [70], hemifacial microsomia, and in a high proportion of craniofacial malformations and syndromes [71]. *Fgf8* is known to play a key role in establishing left-right asymmetry [72], and conditional loss of *Fgf8* in the oral ectoderm of mice [11] and in a hypomorphic zebrafish model [73] result in cranial asymmetry.

### Dynamic expression of *Foxc1* in PA1

Our data indicate that *Foxc1* is initially expressed in the oral ectoderm and cranial mesenchyme contributing to the developing PA1 in E8.5 embryos. At this stage, both *Foxc1* and *Fgf8* are expressed in overlapping domains within the PA1 oral ectoderm **(** **Figures 1A–C** **and** **7A** **)**. In *Foxc1^−/−^* embryos at E8.5, the domain and levels of *Fgf8* are reduced but not absent in the oral ectoderm **(** **Figure 7C** **)**. *Foxc1* therefore is not required to induce *Fgf8* expression in the oral ectoderm, but may be required to maintain proper levels and localization of *Fgf8* **(** **Figure 7** **)**. Conversely, we showed that *Foxc1* activity was reduced in the *Fgf8* allelic series of mutants. Taken together this suggests that *Fgf8* and *Foxc1* potentially form a feedback loop critical for jaw development and in the pathogenesis of syngnathia and TMJ agenesis.

Given the complex and dynamic expression of *Foxc1* in E8.5-10.5 embryos in cranial mesoderm, oral ectoderm and neural crest cell derived PA1 mesenchyme, it will be imperative in the future to delineate the precise requirement for *Foxc1* in each of these tissues during craniofacial growth, patterning and morphogenesis using a repertoire of spatially and temporally specific conditional *Cre* deleter strains. This will shed further light on the spatial and temporal pathogenesis of syngnathia and TMJ agenesis.

### Models of jaw patterning and evolution

Given the important role of jaw development in both evolution and disease, many groups have proposed models to address the concept of polarity within the context of pharyngeal arch development and jaw evolution. Two recent models include the dynamic growth zone model (reviewed in [74]) and the hinge and caps model [75], [76]. Briefly, the growth zone model posits that dorso (proximal)-ventral (distal) polarity of the PA relies upon Edn1 signaling to establish a ventral/distal zone within the PA. Once established, distal *Edn1* and *Bmp4* cues regulate nested *Dlx* gene expression in the PA to establish a combinatorial, dynamic expression code within the arch. The more distal domains contain undifferentiated cell types, while the cells more intermediately and proximally positioned within the mandible differentiate, resolving into zones. The intermediate zone, which expresses *Bapx1*, becomes permissive to forming structures of the jaw joint, and the dorsal/proximal zone is established by *jag1b* expression.

The hinge and caps model [75], [77], [78], places articulation, and subsequently the polarity and modularity, of the upper and lower jaws in the context of cranial neural crest competence to respond to localized epithelial signals. The hinge is defined as the epithelial junction of the maxillary and mandibular prominences of the first arch, also known as the maxillo-mandibular constriction. The caps constitute the lamboidal junction and proximal maxillary region for the upper jaw, and the midline of the mandibular prominences for the lower jaw. Properly patterned placement of the hinge at the sight of articulation and balanced patterning at both caps assures that the elements of the upper and lower jaw will develop in register with one another. Modularity and proximodistal polarity is achieved by the integration of hinge and caps signaling, such that the PA is divided into nested, overlapping developmental fields [75].

The data presented in this study aligns with both the growth zone model and the hinge and caps model. Our data indicate that in the *Foxc1^−/−^* mutant embryos, distal Edn1 and BMP4 signaling within PA1 are normal, and that *Dlx5* and *Dlx3* expression within the recently identified [67] intermediate domain of PA1 is maintained. The loss of a discrete domain of *Dlx5* expression near the maxillo-mandibular constriction in *Foxc1^−/−^* embryos is similar to the disruption reported in *Edn1^fl/fl^;Foxg1-Cre* mutants and provides further evidence of complex control of *Dlx* expression within specific zones of the developing arch [67]. While it is tempting to speculate that disruption of *Foxc1* in our mutants results in a duplication of the condyle or the proximal portion of the dentary rather than a bifurcation, **(** **Figures 3** **and** **6** **)**, we have no molecular data, such as expansion of *Dlx5* or *Dlx6* into the maxillary region, to support this at this time. Additionally, similar to the reported requirement for ectodermal expression of *Edn1* in patterning the intermediate domain, the disruption of *Fgf8* expression in the oral ectoderm of *Foxc1^−/−^* embryos may similarly disrupt *Dlx2* and *Dlx5* in the immediately underlying regions of the PA1 mesenchyme.

With respect to the hinge and caps model, the observed defects in *Foxc1^−/−^* PA1 morphology and skeletal defects affecting the elements involved in jaw articulation (the squamosal, zygomatic complex, and condyle) indicate disruption may center on the proposed hinge region of PA1. In this manner, the expression of both *Foxc1* and *Fgf8* in the PA1 oral ectoderm at E8.5-9.0, in combination with the apparent decrease in *Fgf8* expression localized to the *Foxc1^−/−^* PA1 ectoderm, suggest that *Foxc1* may be required to maintain proper hinge associated signals. Localization of *Dlx2* and *Dlx5* are also primarily altered at the proximal aspect of their expression domains, and the skeletal abnormalities observed are relatively more severe for maxillary jaw elements. Taken together, these may indicate disruption of the symmetric localization of hinge signaling at the maxillo-mandibular constriction in the absence of *Foxc1*. In agreement with the proposed symmetrical alignment of signals at proximal and distal caps, BMP and Edn1 signals remain intact in the *Foxc1^−/−^* embryos and do not display gross evidence of retrognathia **(** **Figure 6** **)**, suggesting the skeletal elements at the cap retain their alignment. This is in contrast to the observed retrognathia when *Edn1* is conditionally disrupted [67], which could be interpreted as altering the distal caps signaling while proximal caps signals remain intact.

Our data indicate that *Foxc1* is required to regulate *Fgf8* activity which influences the localization of *Dlx2* and *Dlx5*. In doing so, *Foxc1* is a key regulator of jaw patterning cues and the elaboration of proximodistal patterning during jaw development. Consequently our studies provide a mechanistic basis for understanding the etiology and pathogenesis of the rare condition of syngnathia and TMJ agenesis. Future studies are still needed, however, to refine the precise spatiotemporal requirement for *Foxc1* in jaw musculoskeletal development. For example, it is unclear whether the abnormal position and size of jaw musclulature in concert with jaw abnormalities reflects a direct requirement for *Foxc1* in pharyngeal arch mesoderm development or is an indirect consequence of abnormal patterning of neural crest cell derived mesenchyme. Previous work has shown that cues from neural crest-derived connective tissue can direct the alignment of mesoderm derived myoblasts [79]–[80], and furthermore that *Dlx* expression in NCCs is required for formation of PA1 derived muscles [66]. This implies that that *Foxc1* maybe predominantly required in neural crest cell derived mesenchyme during jaw musculoskeletal depvelopment. However, our data cannot separate the roles of *Foxc1* in the early cranial mesenchyme, the oral ectoderm, and in the PA1 mesenchymal domain. The recent generation of a conditional *Foxc1* allele [30] and increased understanding of the role of *Foxc1* within neural crest-derived populations, as well as the relationship between abnormally patterned skeletal elements and their associated muscles, will enhance our collective knowledge of jaw development and inform treatment strategies for human patients with syngnathia and other related craniofacial malformations.

## Materials and Methods

### Animal husbandry and genotyping

All mice were housed and all experiments were conducted in compliance with protocols approved by the Institutional Animal Care and Use Committee at the Stowers Institute for Medical Research. *Foxc1* mice were obtained from Tsutomu Kume and were maintained on a 129S6/SvEv background. *Fgf8^Null^* and *Fgf8^Neo^* mice were obtained from Gail Martin and were maintained on a CD1 background. *Z/EG* (stock number 003920, Tg(CAG-Bgeo/GFP)21Lbe/J) and *Wnt1-Cre* (stock number 003829, Tg(Wnt1-cre)11Rth Tg(Wnt1-GAL4)11Rth/J) mice were obtained from the Jackson Laboratory and intercrossed with the *Foxc1* line to generate both *Foxc1;Z/eg* and *Foxc1; Wnt1-Cre* mouse lines. Genotyping of all mouse strains was determined using qPCR with specific probes designed for each strain (Transnetyx, Inc, Cordova, TN, http://www.transnetyx.com). Primer sequences for each assay can be found in **Table S2**.

### Bone and cartilage staining

Early osteoblasts were detected by endogenous alkaline phosphatase activity. At E13.5, heads were bisected, fixed overnight in 4% paraformaldehyde, rinsed in PBS, and then incubated in alkaline phosphatase buffer (100 mM NaCl; 100 mM Tris-HCl, pH 9.5; 50 mM MgCl_2_; 1% Tween-20). Alkaline phosphatase activity was detected using NBT/BCIP. Whole-mount skeletal preparations were made of embryos and neonates (13.5 dpc-P0) as follows. Embryos E15.0 and older were anesthetized by immersion in cold PBS until no reactive movements were seen, the skin and viscera were removed. All embryos were dehydrated in 95% ethanol, transferred to stain base solution (70% ethanol, 5% acetic acid) for 30 minutes, and then stained in 70% ethanol, 5% acetic acid, 0.02% alcian blue, 0.05% alizarin red for 24–48 hours. Following staining, embryos were rinsed in stain base solution, rinsed in water, incubated in 2.0% potassium hydroxide (KOH) (10 minutes to 6 hours based on embryonic stage), and cleared in a 0.25% KOH-glycerol series.

### Histological staining, section and whole-mount *in situ* hybridization

Unless otherwise noted in text, a minimum of three specimens were examined for each genotype. Heads from E16.5 embryos were fixed in 4% paraformaldehyde (PFA) and embedded in O.C.T. compound. 10 µm sections were stained with hematoxylin and eosin following standard procedures. Section *in situ* hybridization was performed with digoxigenin-labeled probes as described in [81]. RNA antisense probes for *Sox9*, *Acan*, *Ihh*, *ColX*, *ColI* and *Scx* were generously provided by Dr. Cliff Tabin. Whole mouse embryos (9.0–10.5 dpc) were fixed overnight in 4% PFA at 4°C, then rinsed in phosphate buffered saline (PBS) with 1% Tween-20 followed by step-wise dehydration to 100% methanol. Anti-sense digoxigenin-labeled (dig-UTP, Roche) riboprobes were synthesized for *Bmp4*, *Dlx2*, *Dlx3*, *Dlx5*, *Dlx6*, *Fgf8*, *Gata3*, *Hand2*, *Msx2*, and *Sox10*. *Foxc1* probe was generated by RT-PCR amplification of a 471 bp fragment spanning the 3′end of exon 1 and a portion of the 3′UTR. Primers: 5′GTACCTGAACCAGGCAGGTG3′, and 5′AGGCAAAAATGGAGGAGGTT3′. Whole mount *in situ* hybridizations were performed according to standard protocols [82], [83] with minor modifications.

### Immunohistochemistry and DAPI staining

Newborn (P0) pups were anesthetized by induction of hypothermia followed by decapitation. (*Foxc1^−/−^* pups were found dead among littermates on P0.) Isolated heads were placed in PBS for several hours and skin was removed prior to fixation for 48 hours at 4°C in 4% PFA. Heads were rinsed in PBS and dehydrated to 80% methanol, and were stored for at least one week in Dent's fixative at 4°C. Endogenous alkaline phosphatase activity was inactivated by incubation in Dent's bleach prior to rehydration into PBS containing 0.1% Tween-20 (PBST). Heads were incubated overnight in PBST, 10% fetal bovine serum (PBST-FBS) followed by incubation in alkaline phosphatase conjugated anti-Myosin (MY-32, Sigma, A4335), diluted to 10 µg/ml in PBST-FBS for 24 hours at 4°C. Heads were washed in PBST and alkaline phosphatase was detected with NBT/BCIP.

Neurofilament whole mount immunostaining was performed on 4% PFA fixed E11.5 embryos. Endogenous peroxidase activity was blocked by incubation in Dent's bleach overnight at room temperature. Embryos were rehydrated to PBST, blocked in 20% goat serum in PBST, and incubated in 1∶500 dilution of 2H3 antibody (Developmental Studies Hybridoma Bank) overnight at room temperature. Embryos were rinsed in PBST, and then incubated with a 1∶200 dilution of a horseradish peroxidase (HRP) conjugated donkey anti-mouse IgG secondary antibody (Jackson ImmunoResearch, 715-035-150). Signal was detected through diaminobenzidene (DAB) staining (Sigma, D5905). Stained embryos were cleared in 20% glycerol, 0.25% KOH prior to imaging.

For sectional immunohistochemistry to detect muscle actin (HHF35, Dako, M0635), E17.5 embryos were dissected, anesthetized by immersion in cold PBS, and then decapitated. Heads were fixed in 4% PFA, dehydrated to 100% ethanol, and embedded in paraffin. Frontal and sagittal sections were cut (10 µm). Sections were dewaxed and rehydrated to PBS. HHF35 was biotinylated with the MM Biotinylation Kit (Biocare Medical, MMBK G,H) and then applied to sections at a 1∶100 dilution overnight at 4°C. Following streptavidin-HRP incubation, muscle actin reactivity was detected with DAB staining. Sections were counterstained with 10% hematoxylin.

For gross visualization of palate and tongue tissues, upper and lower jaws were separated and incubated overnight in DAPI (2 µg/ml). Tissues were imaged on a Leica MZ FLIII stereoscope using Axiovision software. Similarly, whole embryos between E9.0-11.5 were fixed in 4% PFA, rinsed in PBS, and incubated in DAPI. Embryos were then cleared in 50% glycerol, mounted with Vectashield (Vector Labs, H1000), and scanned using a Zeiss LSM5 Upright Pascal Confocal microscope. Projected Z-stacks were flattened and exported to Adobe Photoshop.

### β-galactosidase staining

Whole embryos from E8.5-11.5 were collected and fixed from 30 to 60 minutes in 0.2% glutaraldehyde, 5 µM EGTA, 100 µM MgCl_2_ on ice. Embryos were rinsed and stained according to manufacturer's protocol (Millipore #BG-6-B, #BG-7-B, #BG-8-C).

### Apoptosis and proliferation assays

Embryos (E8.75 - 9.5) were fixed overnight in 4% PFA at 4°C. Embryos were rinsed in PBS and apoptosis was detected in whole embryo samples by TUNEL labeling using the *In Situ* Cell Death Detection Kit (Roche) for 4 hours at 37°C. Embryos were then counterstained in DAPI, mounted in Vectashield, and scanned using a Zeiss LSM5 Upright Pascal Confocal microscope. Projected Z-stacks were pseudocolored using Zeiss AIM software, flattened, and exported to Adobe Photoshop. Proliferation was assayed by immunohistochemistry for phosphohistone H3 (Upstate, 06-570,1∶500 dilution) on cryosections of E9.0 mouse embryos. Secondary antibody was AlexaFluor 488 conjugated goat anti-rabbit IgG (Invitrogen, A11034, 1∶300 dilution). Sections containing pharyngeal arch one were photographed on a Zeiss Axioplan compound microscope using Axiovision software. Phosphohistone H3 positive cells and DAPI stained nuclei within PA1 were counted using Image J to determine a mitotic index for PA1. An average mitotic index and standard deviation was calculated for both wild-type and mutant embryos. Statistical significance of differences between wild-type and *Foxc1^−/−^* counts was assessed using an unpaired, two-tailed Student's t-test with significance taken at p≤0.05.

### Determination of cell cycle length

S-phase and cell cycle length were analyzed by incorporation of IdU-BrdU as previously described [36]. Pregnant females were injected intraperitoneally with IdU at 0.1 mg/kg body weight. After 1.5 hours, mice were injected intraperitoneally with 0.1 mg/kg body weight of BrdU. Two hours after IdU injection, mice were euthanized and embryos collected. Cryosections were then prepared and the IdU and BrdU positive cells were detected by immunostaining using mouse anti-BrdU antibody (BD Bioscience, which recognizes both IdU and BrdU) and rat anti-BrdU antibody (Abcam, which recognizes BrdU only). Counts were conducted on 4 non-adjacent sections of the cranial mesenchyme (5–7 s), PA1 mesenchyme (10–13 s), and oral ectoderm for each specimen. Cell cycle length was calculated as described previously [36].

### qPCR

The cranial region of E8.5 embryos was isolated by using glass needles to cut transversely just anterior to the heart. For E9.5 embryos, cuts were made posterior and dorsl to PA1 and transversely at the level of the developing eye in order to isolate PA1. For each genotype, 3 pools of total RNA (dissected tissue from 3 embryos per pool) were isolated using the Qiagen RNeasy Mini Kit. Between 250–400 ng of total RNA from each pool was then used to generate random primed single-stranded cDNA (Superscript RTIII First Strand cDNA Synthesis Kit, Invitrogen). Relative levels of mRNA were determined using a qRT-PCR PowerSYBR (Applied Biosystems) assay with the following primers: *Fgf8:* 5′AATCCAGCCCCAAACTACC3′ and 5′GCTCTGCTCCCTCACATG3′; *Foxc1:* 5′TTCTTGCGTTCAGAGACTCG3′ and 5′AGGTACTTTCCCGTTCTTTCG3′ and internal control primers for *Atp5b*, *Canx*, *Gapdh,* and *Ubc*.

### Imaging of whole-mount specimens and image processing

Whole mount embryos were photographed using a Leica MZ16 stereoscope, Nikon Digital Sight DS-Ri1 camera, and Nikon NIS Elements BR 3.2 software, unless otherwise noted. A subset of the images (Figure 1A, B, D, F; Figure 2; Figure 5A–D, M–P; Figure 6A, C, E–F, H–J, L–R; Figure S3A–B, I–J, Figure S7A–C, F,G, I, J) was acquired as a manual series of Z-stacks. These images were further processed using Helicon Focus (Helicon Soft, Ltd, http://www.heliconsoft.com) to compile and render the focused regions of the multiple focal planes into a single in focus image.

## Supporting Information

Figure S1 Histological analysis of TMJ abnormalities in *Foxc1^−/−^* embryos. (A) Different cellular zones in the E17.5 wild-type condyle are indicated. (B–G) Representative serial coronal cryosections of E17.5 wild type (B, D, F) and mutant (C, E, G) heads. (B, C) Hematoxylin and eosin stained sections show normal anatomy of the condyle (cdp), glenoid fossa (f), and joint disc (d) separating upper and lower synovial joint cavities in *Foxc1^+/+^* embryos. In *Foxc1^−/−^* embryos, the fossa and disc are absent, and the condyle is bifurcated (cdp*). (D–G) To aid visualization muscles are outlined in red, and the trigeminal ganglion (trg), bone, and cartilage elements of the TMJ are outlined in black. (F) Magnification of selected area in (D) showing the *Foxc1^+/+^* TMJ and associated temporalis (tm), lateral pterygoid (lp), and medial pterygoid (mp) muscles. The angular process (agp) and Meckel's cartilage (MC) are well formed. (G) Magnification of the selected area in (E) showing the *Foxc1^−/−^* TMJ. The bifurcated condyle (cdp *) and MC can clearly be distinguished. The temporalis muscle is shifted to occupy the space where the squamosal and fossa are found in the wild type TMJ. The orientation of the muscles (tm, lp, and mp) is altered in mutants compared to controls. Magnification is 10× (A,B, C),4× (D, E), and 6× (F, G). Abbreviations: fc, flattened chondrocytes; fl, fibrous cell layer; hc, hypertrophic chondrocytes; pcl, progenitor cell layer; pe, perichondrium.
(TIF)

Figure S2 Mild palatal defects in absence of *Foxc1*. (A, C) Ventral view of wild-type (A) and *Foxc1^−/−^* (C) P0 skeletal palatal elements. The palatine (pl) and pterygoids (ptg) are small and a small cleft is seen in the palatal process of the palatine (pppl) in the mutant. The mutant basisphenoid (bs) remains open at the midline. (B, D) Gross view of *Foxc1^+/+^* (B) and *Foxc1^−/−^* (C) palates. No cleft is seen in the soft tissue of the mutant, but the rugae (r) are less sharply delineated and fewer in number than in wild-type. (E, F) Dorsal view of wild-type (E) and mutant (F) tongue. Fungiform (fp) and median circumvallate (mcp) papillae form in the absence of *Foxc1*. However, the anterior portion of the tongue (atg) is spade shaped, possibly due to constriction of the posterior portion of the tongue by the syngnathic jaw. Scale bars: 500 µm Abbreviations: ip, incisive papilla; ppmx, palatal process of maxilla.
(TIF)

Figure S3 Normal neural crest formation, migration, lineage contribution, and peripheral nervous system patterning in *Foxc1^−/−^* embryos. (A, B) Whole mount *in situ* hybridization for *Sox10* shows normally migrating cranial neural crest cells in *Foxc1^+/+^* (A) and *Foxc1^−/−^* (B) embryos at E9.0 (C–F) Whole mount images of freshly dissected *Foxc1*; *Wnt1Cre*; *Z/EG* embryos indicate normal contribution of neural crest derived cells (GFP, green) to cranial regions of both *Foxc1^+/+^* (C, E) and *Foxc1^−/−^* (D, F) embryos at E10.5 (C, D) and E12.5 (E, F). (G, H) Whole mount immuno-detection of neurofilament at E11.5 reveals normal formation and patterning of cranial nerves V, VII, VIII, IX, and X in wild type (G) and mutant (H) embryos. Scale bars: (A, B) 200 µm; (C–H) 500 µm. Abbreviations: mab, mandibular branch of trigeminal nerve; mxb, maxillary branch of trigeminal nerve; op, ophthalmic branch of trigeminal nerve.
(TIF)

Figure S4 Normal cell proliferation rate and apoptosis in *Foxc1^−/−^* PA1. (A, B) Representative sagittal cryosections through E9.0 *Foxc1^+/+^* (A) and *Foxc1^−/−^* (B) PA1 immunostained for phosphohistone H3 (pH 3) (green) and counterstained with DAPI (blue). For each section, all DAPI stained nuclei were counted as were the pH 3 positive nuclei. The white line in each section delimits the region containing the pharyngeal arch in which nuclei were counted. (C) Quantification of mitotic index in *Foxc1^+/+^* and *Foxc1^−/−^* PA1s. No significant difference was found between control and mutant (p = 0.116). Scale bars: 100 µm.
(TIF)

Figure S5 Distal patterning cues are normally expressed in *Foxc1^−/−^*. Whole mount *in situ* hybridization in *Foxc1^+/+^* (A, C, E, G, I, K, M, O) and *Foxc1^−/−^* (B, D, F, H, J, L, N, P) E10.5 embryos. Lateral (A, B, E, F, I, J, M, N) and frontal (C, D, G, H, K, L, O, P) views are shown for each probe. (A–D) *Bmp4* and its target *Msx2* (E–H) are normally expressed in the nasal prominence epithelia, maxillary prominence, and distal mandibular ectoderm in both control and mutant embryos. (I–L) *Hand2*, a downstream target of endothelin-1 signaling, is normally expressed in the distal PA1 mesenchyme in *Foxc1^+/+^* and *Foxc1^−/−^* embryos. (M–P) *Gata3*, which is required for an endothelin-A receptor independent expression of *Hand2*, is also normally expressed in the absence of *Foxc1*. Scale bars: 500 µm.
(TIF)

Figure S6 Middle ear, palatal abnormalities, and facial asymmetry in compound *Fgf8* and *Foxc1;Fgf8* mutants. (A–C) Dissected middle ear ossicles from P0 *Foxc1^+/+^* (A), *Foxc1^−/−^* (B), and *Foxc1^−/−^;Fgf8^Null/+^* (C) mice. The wild type malleus (mal) and incus (in) show regions of ossification (red), whereas the mutant ossicles are hypoplastic and remain as cartilage. The goniale (gn) ossifies, but is smaller in the mutants. The stapes (st) is formed in wild type, but is absent from mutants. (D–F) Alcian blue (cartilage) and alizarin red (bone) stained skeletal preparations showing palatal elements of *Fgf8^+/+^* (D), *Fgf8^Null/Neo^* (E), *Foxc1^−/−^;Fgf8^Null/+^* (F) embryos at E17.5. (E) In *Fgf8^Null/Neo^* embryos, the palatal process of the maxilla (ppmx) and palatal process of the palatine (pppl) are smaller than controls (D). The pterygoids (ptg) and the basisphenoid (bs) are misshapen. (F) In *Foxc1^−/−^;Fgf8^Null/+^* embryos, the palatal processes (ppmx, pppl) are not formed. The pterygoids are hypoplastic and only small lateral portions of the basisphenoid form. (G–I) Frontal view of upper and lower jaw elements in *Fgf8^+/+^* (G), *Fgf8^Null/Neo^* (H), *Foxc1^−/−^;Fgf8^Null/+^* (I) embryos at E17.5. In both compound mutants, the dosage of *Fgf8* is genetically reduced and asymmetry is observed in the skeletal elements of the jaw. (J) Gross frontal view of *Foxc1^−/−^;Fgf8^Null/Neo^* fetus showing overt facial asymmetry. Scale bars: (A–C) 200 µm; (D–J) 500 µm Abbreviations: dnt, dentary; fmx, frontal process of the maxilla; mm, manubrium; pb, processus brevus; sym, mandibular symphisis.
(TIF)

Table S1 Case reports of human bony syngnathia. Summary of published cases of syngnathia from 1936–2011 highlighting their characteristic features and isolated versus syndromic occurrence.
(DOC)

Table S2 Genotyping primer sequences. Summary of the sequences used for genotyping and RT-PCR.
(DOC)
