# Supplementary figures and images for: Interaction between Foxc1 and Fgf8 during Mammalian Jaw Patterning and in the Pathogenesis of Syngnathia
Source: PLoS Genet. 2013 Dec 19;9(12):e1003949. doi: 10.1371/journal.pgen.1003949 (PMC3868537; doi:10.1371/journal.pgen.1003949)

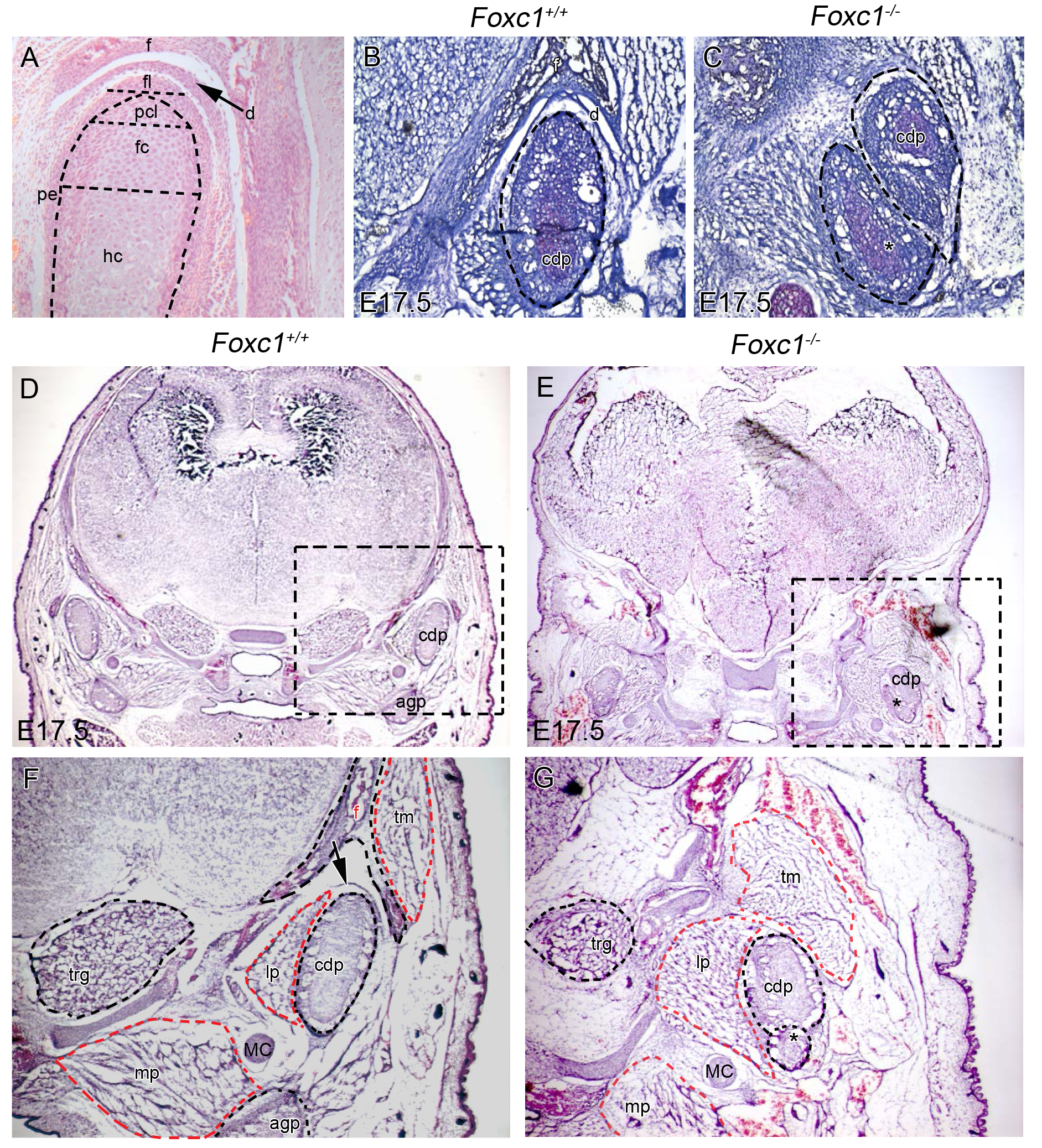

Supplement: Figure S1 — Histological analysis of TMJ abnormalities in Foxc1−/− embryos. (A) Different cellular zones in the E17.5 wild-type condyle are indicated. (B–G) Representative serial coronal cryosections of E17.5 wild type (B, D, F) and mutant (C, E, G) heads. (B, C) Hematoxylin and eosin stained sections show normal anatomy of the condyle (cdp), glenoid fossa (f), and joint disc (d) separating upper and lower synovial joint cavities in Foxc1+/+ embryos. In Foxc1−/− embryos, the fossa and disc are absent, and the condyle is bifurcated (cdp*). (D–G) To aid visualization muscles are outlined in red, and the trigeminal ganglion (trg), bone, and cartilage elements of the TMJ are outlined in black. (F) Magnification of selected area in (D) showing the Foxc1+/+ TMJ and associated temporalis (tm), lateral pterygoid (lp), and medial pterygoid (mp) muscles. The angular process (agp) and Meckel's cartilage (MC) are well formed. (G) Magnification of the selected area in (E) showing the Foxc1−/− TMJ. The bifurcated condyle (cdp *) and MC can clearly be distinguished. The temporalis muscle is shifted to occupy the space where the squamosal and fossa are found in the wild type TMJ. The orientation of the muscles (tm, lp, and mp) is altered in mutants compared to controls. Magnification is 10× (A,B, C),4× (D, E), and 6× (F, G). Abbreviations: fc, flattened chondrocytes; fl, fibrous cell layer; hc, hypertrophic chondrocytes; pcl, progenitor cell layer; pe, perichondrium. (TIF) [file pgen.1003949.s001.tif]

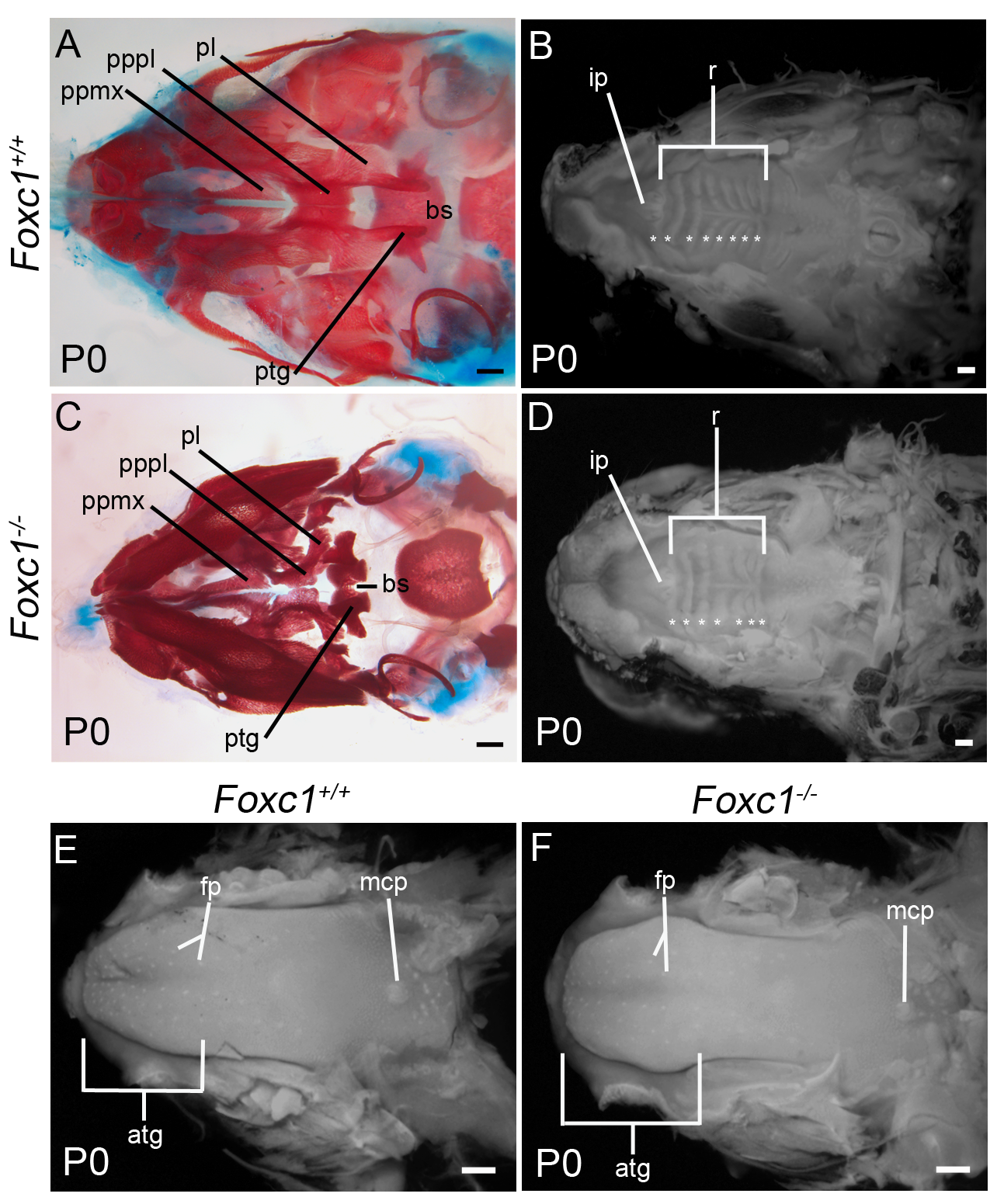

Supplement: Figure S2 — Mild palatal defects in absence of Foxc1. (A, C) Ventral view of wild-type (A) and Foxc1−/− (C) P0 skeletal palatal elements. The palatine (pl) and pterygoids (ptg) are small and a small cleft is seen in the palatal process of the palatine (pppl) in the mutant. The mutant basisphenoid (bs) remains open at the midline. (B, D) Gross view of Foxc1+/+ (B) and Foxc1−/− (C) palates. No cleft is seen in the soft tissue of the mutant, but the rugae (r) are less sharply delineated and fewer in number than in wild-type. (E, F) Dorsal view of wild-type (E) and mutant (F) tongue. Fungiform (fp) and median circumvallate (mcp) papillae form in the absence of Foxc1. However, the anterior portion of the tongue (atg) is spade shaped, possibly due to constriction of the posterior portion of the tongue by the syngnathic jaw. Scale bars: 500 µm Abbreviations: ip, incisive papilla; ppmx, palatal process of maxilla. (TIF) [file pgen.1003949.s002.tif]

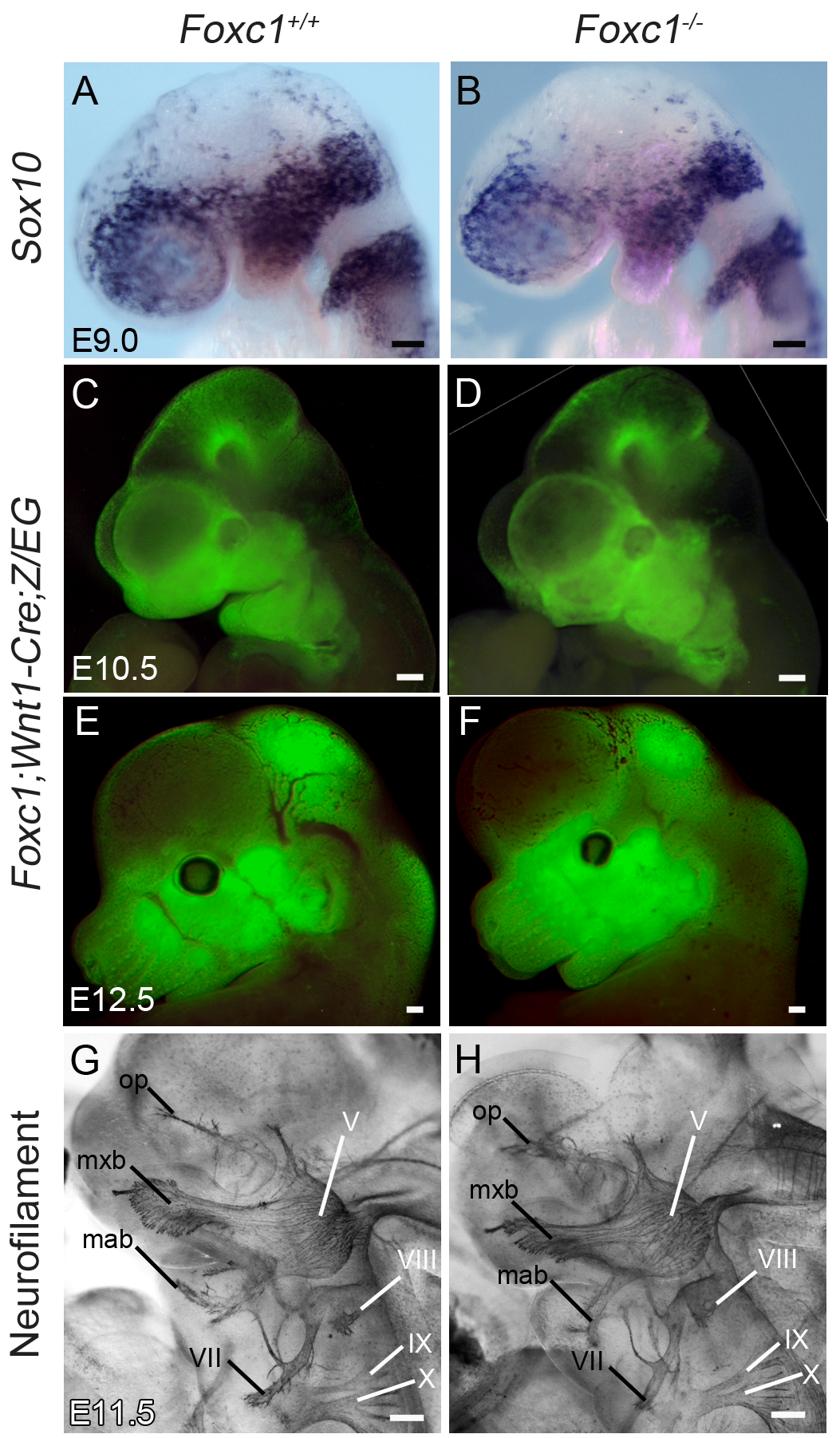

Supplement: Figure S3 — Normal neural crest formation, migration, lineage contribution, and peripheral nervous system patterning in Foxc1−/− embryos. (A, B) Whole mount in situ hybridization for Sox10 shows normally migrating cranial neural crest cells in Foxc1+/+ (A) and Foxc1−/− (B) embryos at E9.0 (C–F) Whole mount images of freshly dissected Foxc1; Wnt1Cre; Z/EG embryos indicate normal contribution of neural crest derived cells (GFP, green) to cranial regions of both Foxc1+/+ (C, E) and Foxc1−/− (D, F) embryos at E10.5 (C, D) and E12.5 (E, F). (G, H) Whole mount immuno-detection of neurofilament at E11.5 reveals normal formation and patterning of cranial nerves V, VII, VIII, IX, and X in wild type (G) and mutant (H) embryos. Scale bars: (A, B) 200 µm; (C–H) 500 µm. Abbreviations: mab, mandibular branch of trigeminal nerve; mxb, maxillary branch of trigeminal nerve; op, ophthalmic branch of trigeminal nerve. (TIF) [file pgen.1003949.s003.tif]

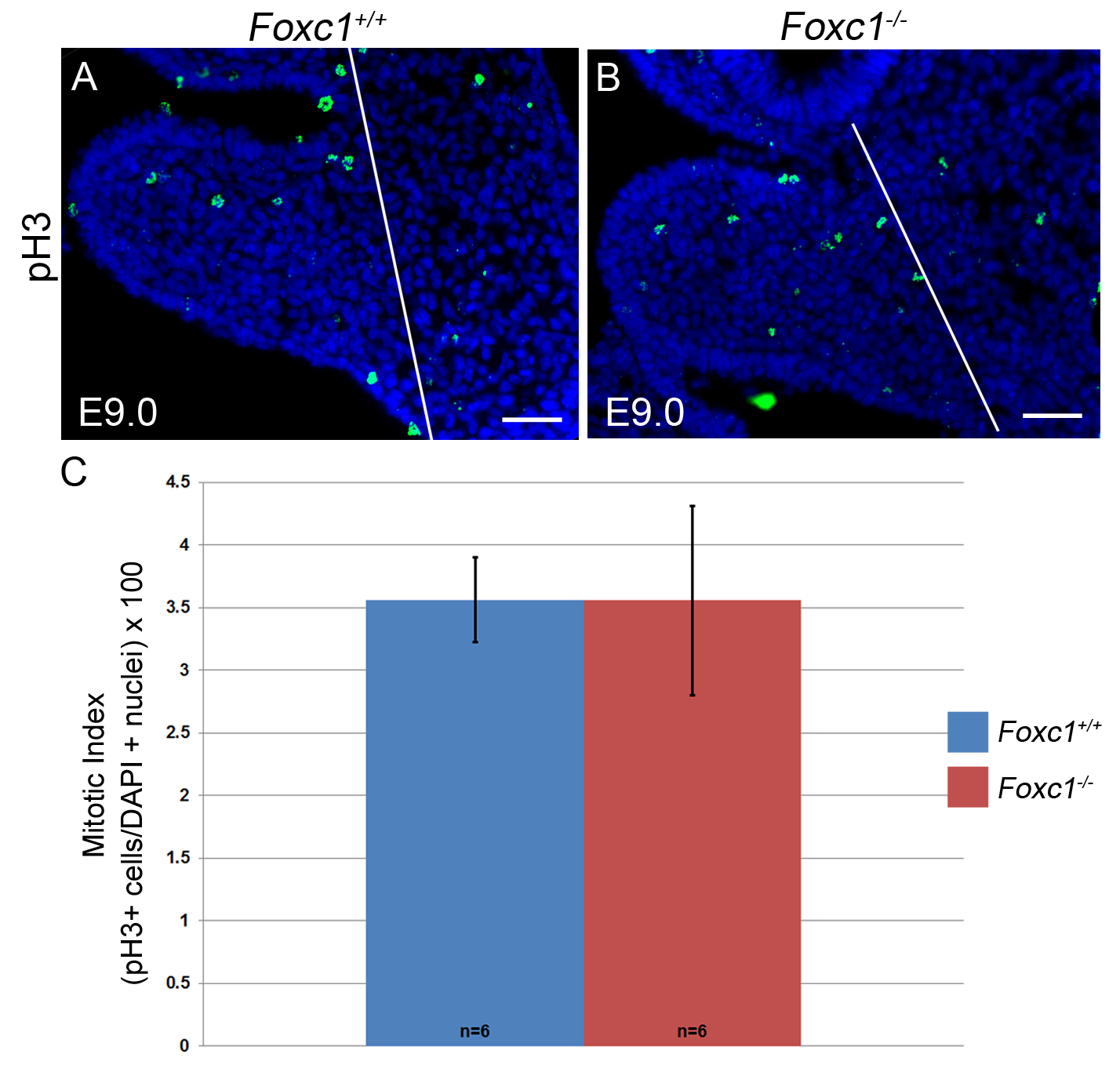

Supplement: Figure S4 — Normal cell proliferation rate and apoptosis in Foxc1−/− PA1. (A, B) Representative sagittal cryosections through E9.0 Foxc1+/+ (A) and Foxc1−/− (B) PA1 immunostained for phosphohistone H3 (pH 3) (green) and counterstained with DAPI (blue). For each section, all DAPI stained nuclei were counted as were the pH 3 positive nuclei. The white line in each section delimits the region containing the pharyngeal arch in which nuclei were counted. (C) Quantification of mitotic index in Foxc1+/+ and Foxc1−/− PA1s. No significant difference was found between control and mutant (p = 0.116). Scale bars: 100 µm. (TIF) [file pgen.1003949.s004.tif]

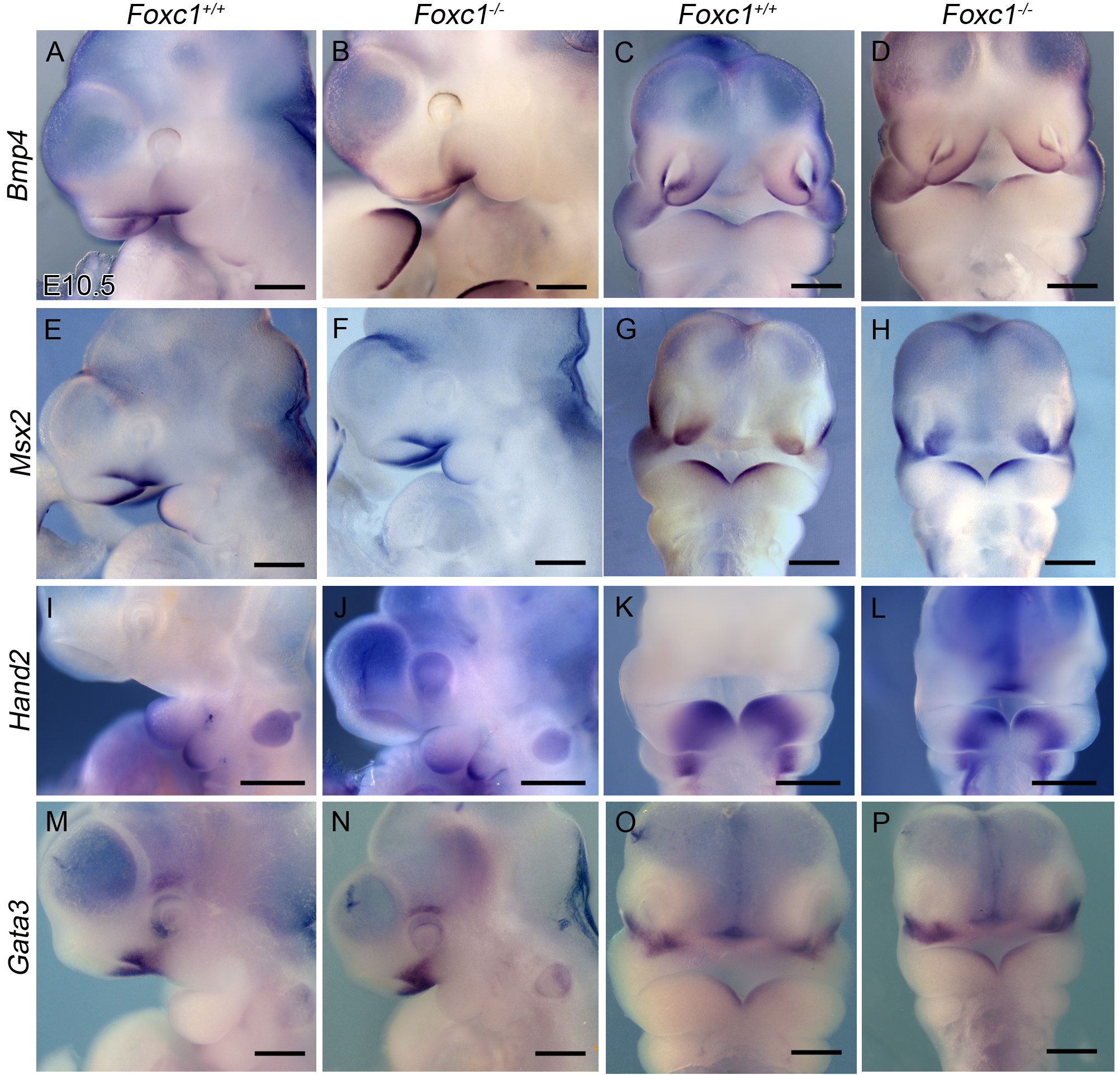

Supplement: Figure S5 — Distal patterning cues are normally expressed in Foxc1−/−. Whole mount in situ hybridization in Foxc1+/+ (A, C, E, G, I, K, M, O) and Foxc1−/− (B, D, F, H, J, L, N, P) E10.5 embryos. Lateral (A, B, E, F, I, J, M, N) and frontal (C, D, G, H, K, L, O, P) views are shown for each probe. (A–D) Bmp4 and its target Msx2 (E–H) are normally expressed in the nasal prominence epithelia, maxillary prominence, and distal mandibular ectoderm in both control and mutant embryos. (I–L) Hand2, a downstream target of endothelin-1 signaling, is normally expressed in the distal PA1 mesenchyme in Foxc1+/+ and Foxc1−/− embryos. (M–P) Gata3, which is required for an endothelin-A receptor independent expression of Hand2, is also normally expressed in the absence of Foxc1. Scale bars: 500 µm. (TIF) [file pgen.1003949.s005.tif]

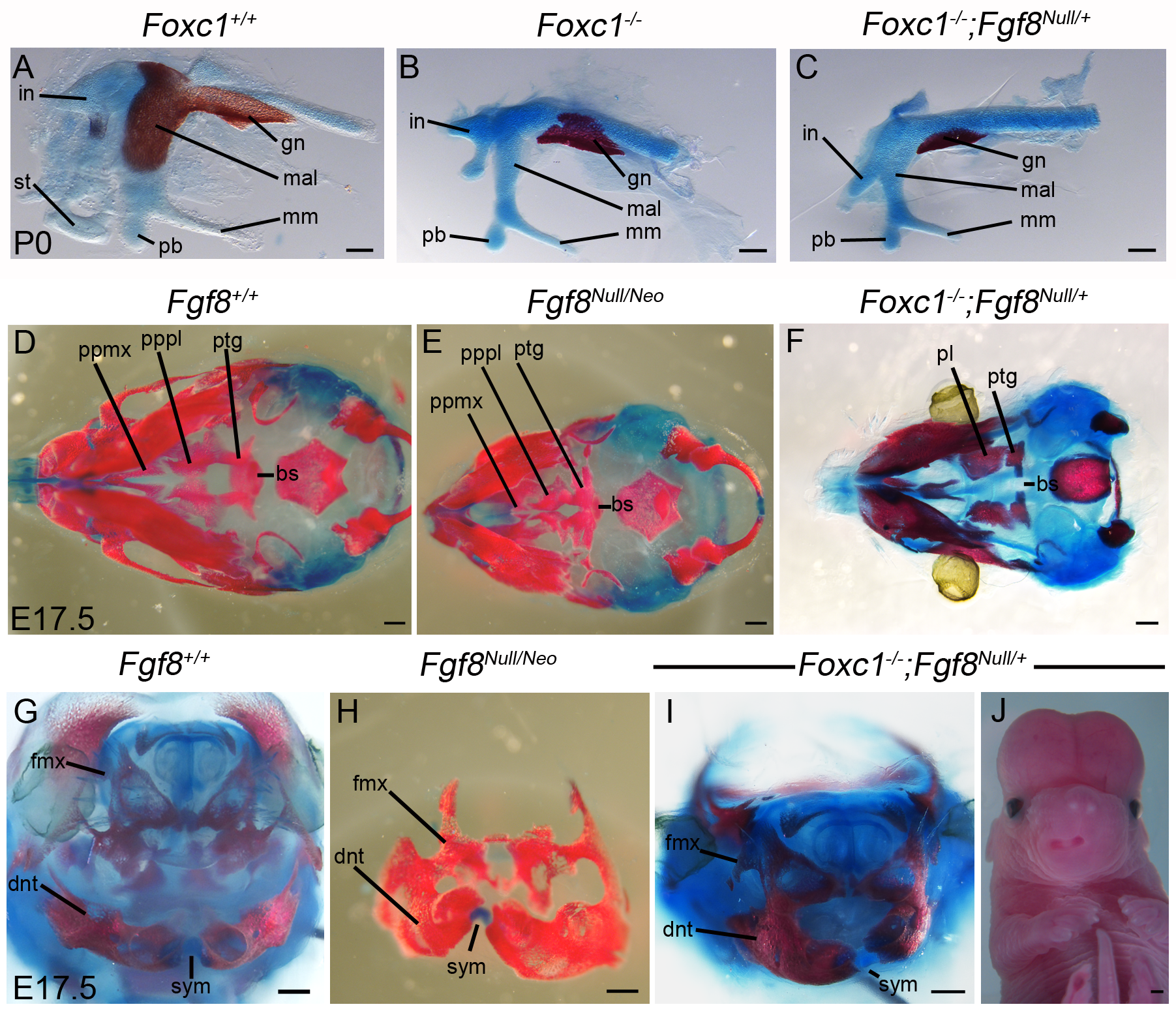

Supplement: Figure S6 — Middle ear, palatal abnormalities, and facial asymmetry in compound Fgf8 and Foxc1;Fgf8 mutants. (A–C) Dissected middle ear ossicles from P0 Foxc1+/+ (A), Foxc1−/− (B), and Foxc1−/−;Fgf8Null/+ (C) mice. The wild type malleus (mal) and incus (in) show regions of ossification (red), whereas the mutant ossicles are hypoplastic and remain as cartilage. The goniale (gn) ossifies, but is smaller in the mutants. The stapes (st) is formed in wild type, but is absent from mutants. (D–F) Alcian blue (cartilage) and alizarin red (bone) stained skeletal preparations showing palatal elements of Fgf8+/+ (D), Fgf8Null/Neo (E), Foxc1−/−;Fgf8Null/+ (F) embryos at E17.5. (E) In Fgf8Null/Neo embryos, the palatal process of the maxilla (ppmx) and palatal process of the palatine (pppl) are smaller than controls (D). The pterygoids (ptg) and the basisphenoid (bs) are misshapen. (F) In Foxc1−/−;Fgf8Null/+ embryos, the palatal processes (ppmx, pppl) are not formed. The pterygoids are hypoplastic and only small lateral portions of the basisphenoid form. (G–I) Frontal view of upper and lower jaw elements in Fgf8+/+ (G), Fgf8Null/Neo (H), Foxc1−/−;Fgf8Null/+ (I) embryos at E17.5. In both compound mutants, the dosage of Fgf8 is genetically reduced and asymmetry is observed in the skeletal elements of the jaw. (J) Gross frontal view of Foxc1−/−;Fgf8Null/Neo fetus showing overt facial asymmetry. Scale bars: (A–C) 200 µm; (D–J) 500 µm Abbreviations: dnt, dentary; fmx, frontal process of the maxilla; mm, manubrium; pb, processus brevus; sym, mandibular symphisis. (TIF) [file pgen.1003949.s006.tif]
