# Supplementary material for: Interaction between Foxc1 and Fgf8 during Mammalian Jaw Patterning and in the Pathogenesis of Syngnathia
Source: PLoS Genet. 2013 Dec 19;9(12):e1003949. doi: 10.1371/journal.pgen.1003949 (PMC3868537; doi:10.1371/journal.pgen.1003949)
Supplement: Table S1 — Case reports of human bony syngnathia. Summary of published cases of syngnathia from 1936–2011 highlighting their characteristic features and isolated versus syndromic occurrence. (DOC) [file pgen.1003949.s007.doc]

Table S1: Case reports of human bony syngnathia.

| **Year** | **Author(s)** | **Site of Fusion^a^** | **Cleft Palate** | **Tongue** | **TMJ** | **Isolated** | **Associated Syndrome** | **Comments** |
| --- | --- | --- | --- | --- | --- | --- | --- | --- |
| 1936 | Burket, LW | proximal, unilateral | no | normal | normal |  | hemifacial microsomia |  |
| 1948 | Hochstetter, F | alveolar, bilateral |  | normal | normal | yes |  | Pictured in Goodacre, TE and Wallace, AF, 1990 |
| 1961 | Patterson, G | distal midline | yes | aglossia | normal |  | aglossia adactylia |  |
| 1965 | Salleh, NM | alveolar, unilateral | no | normal | normal | yes |  |  |
| 1966 | Snijman, PC et al. | alveolar, bilateral | no | slight hypoglossia | abnormal |  |  |  |
| 1969 | Hoggins, GS | distal midline | yes | aglossia | normal |  | aglossia adactylia |  |
| 1971 | Bernard, R et al. | alveolar, bilateral | no | aglossia |  |  | aglossia adactylia |  |
| 1976 | Alvarez, GE | complete, bilateral | yes | aglossia | normal |  | aglossia adactylia |  |
| 1977 | Shah, RM | alveolar, bilateral | yes | hypoglossia | normal |  | unidentified | 8 week human fetus |
| 1979 | Miskinyar, SA | alveolar, unilateral | no | normal | normal | yes |  |  |
| 1983 | Dobrow, B | complete, bilateral | severe palate malform-ation | aglossia | absent |  | unidentified | coloboma |
| 1983 | Kittur, SD et al. | proximal, unilateral | yes | hypoglossia | abnormal |  | unidentified |  |
| 1985 | Simpson, JR and Maves, MD | complete, bilateral |  |  |  |  |  |  |
| 1986 | Nwoku, AL and Kekere-Ekun, TA | complete, bilateral | no | normal | normal | yes |  |  |
| 1990 | Brown, DM and Marsh, JL | proximal, bilateral | no | normal | absent |  | unidentified |  |
| 1990 | Goodacre, TE and Wallace, AF | alveolar, bilateral | no | normal | normal |  | unidentified | case 2 |
| 1990 | Goodacre, TE and Wallace, AF | alveolar, bilateral |  |  |  |  | unidentified | case 4 |
| 1990 | Traaholt, LM | alveolar, unilateral | yes | normal | normal |  | unidentified |  |
| 1990 | Domarus, H and Scheunemann, H | proximal, bilateral | no | normal | normal | yes |  | case 1 |
| 1990 | Domarus, H and Scheunemann, H | proximal, bilateral | no | normal | normal | yes |  | case 2 |
| 1993 | Gartlan, MG et al. | distal midline | yes | hypoglossia | normal |  | unidentified | case 2 |
| 1993 | Agarwal, K et al. | alveolar, bilateral | no | normal | normal | yes |  |  |
| 1994 | Arshad, AR and Goh, CS | alveolar, bilateral | no | hypoglossia | normal |  | unidentified | case 1 |
| 1994 | Arshad, AR and Goh, CS | alveolar, bilateral | no | hypoglossia | normal |  | aglossia adactylia | case 2 |
| 1996 | Kamata, S et al. | complete, bilateral | no | normal | absent |  | unidentified |  |
| 1996 | Behnia, H and Shamse, ME | complete, unilateral | no | normal | abnormal, fused to temporal bone | yes |  |  |
| 1996 | Jackson, IT et al. | proximal, bilateral | yes | normal | normal |  | unidentified |  |
| 1997 | Dawson, KH et al. | palato-mandibular, bilateral | yes | aglossia | abnormal |  | aglossia adactylia | case 1 |
| 1997 | Dawson, KH et al. | alveolar, unilateral | no | normal | normal |  | unidentified | case 2 |
| 1997 | Dawson, KH et al. | alveolar, unilateral | no | normal | absent |  | hemifacial microsomia | case 3 |
| 1997 | Dawson, KH et al. | alveolar, unilateral | yes | normal | absent |  | hemifacial microsomia | case 4 |
| 1997 | Dawson, KH et al. | proximal, bilateral | yes | normal | absent |  | unidentified | case 5 |
| 1997 | Rao, S, et al. | palato-mandibular, midline | no | bifid | normal |  | unidentified |  |
| 1999 | Ugurlu, K et al. | complete, bilateral | no | slight hypoglossia | absent |  | unidentified |  |
| 2000 | Yazdi, I and Fakhraee, H | proximal, bilateral | no | normal | normal | yes |  |  |
| 2000 | Knoll, B et al. | alveolar, bilateral | yes | aglossia | normal |  | aglossia adactylia |  |
| 2001 | Laster, Z et al. | proximal, bilateral | yes | normal | normal |  | unidentified |  |
| 2004 | Verloes, A et al. | complete, unilateral | yes | normal | reduced |  | unidentified |  |
| 2004 | Daniels, JS | proximal, bilateral | no | normal | normal | yes |  |  |
| 2005 | Ugurlu, K et al. | proximal, bilateral | no | normal | absent |  | unidentified |  |
| 2006 | Shams, MG et al. | proximal, unilateral | no | normal | normal | yes |  | bilateral fibrous adhesions |
| 2006 | Nikolic, S. et al. | proximal, unilateral | no | normal | abnormal, fused | yes |  |  |
| 2007 | Mir et al. | alveolar, bilateral | no | normal | normal | yes |  |  |
| 2007 | Trigg, DJ et al. | proximal, bilateral | no | normal | normal | yes |  |  |
| 2007 | Vahidi, K et al. | proximal, bilateral | no | normal | normal | yes |  |  |
| 2008 | Gupta, RK et al. | alveolar, bilateral | no | normal | normal | yes |  |  |
| 2010 | Konas, E et al. | proximal, unilateral | yes | normal | abnormal |  | unidentified |  |
| 2010 | El-Hakim, IE et al. | proximal, bilateral | no | normal | normal | yes |  |  |
| 2010 | Fallahi, HR | complete, unilateral | no | normal | normal |  | unidentified |  |
| 2010 | Halli, R. et al. | proximal, unilateral | no | normal | reduced |  | unidentified |  |
| 2010 | Naikmasur, VG et al. | alveolar, unilateral | no | normal | ankylosed | yes |  | 60 year old woman |
| 2010 | Subramanian, B et al. | proximal, bilateral | no | normal | abnormal | yes |  | case1 |
| 2010 | Subramanian, B et al. | proximal (at TMJ), bilateral | no | normal | abnormal, fused | yes |  | case2 |
| 2010 | Subramanian, B et al. | proximal, bilateral | unknown | unknown | unknown |  |  | case3 |
| 2010 | Subramanian, B et al. | distal midline | no | normal | normal |  | oral mandibular limb hypogenesis | case4 |
| 2011 | Tauro, DP et al. | proximal, unilateral | no | normal | normal |  |  | right anophthalmia |

^a^Site of Fusion – Proximal: fusion near the site of articulation, usually between the zygomatic complex and the dentary. Alveolar: fusion in region of tooth development. Complete: fusion in both proximal and alveolar regions.

**Supplementary References**

1. Burket LW (1936) Congenital bony temporomandibular ankylosis and facial hemiatrophy. Review of the literature and report of a case. JAMA 106: 1719-1722.

2. Hochstetter F (1948) Uber zwei Falle epithelialer syngnathie bei menschlichen Keimlingen. Deutsche Medizinische Wochenschrift 108: 1.

3. Goodacre TE, Wallace AF (1990) Congenital alveolar fusion. British Journal Of Plastic Surgery 43: 203-209.

4. Patterson G (1961) Aglossia congenita with bony fusion of the jaw. Acta Chirug Scand 122: 93-95.

5. Salleh NM (1965) Congenital partial fusion of the maxilla and mandible. Oral Surgery, Oral Medicine, Oral Pathology 20: 74-76.

6. Snijman PC, Prinsloo JG (1966) Congenital fusion of the gums. Amer J Dis Child 112: 593-595.

7. Hoggins GS (1969) Aglossia congenita with bony fusion of the jaws. British Journal of Oral Surgery 7: 63-65.

8. Bernard R, Lachard J, Garcin M, Romette JM, Gola R, et al. (1971) A case of aglosso-adactylia with anterior bone fusion of the jaws. Rev Stomatol Chir Maxillofac 72: 315-320.

9. Alvarez GE (1976) The aglossia-adactylia syndrome. British Journal Of Plastic Surgery 29: 175-178.

10. Shah RM (1977) Palatomandibular and maxillo-mandibular fusion, partial aglossia, and cleft palate in a human embryo. Report of a case. Teratology 15: 261-272.

11. Miskinyar SAC (1979) Congenital mandibulomaxillary fusion. Plastic And Reconstructive Surgery 63: 120.

12. Dobrow B (1983) Syngnathia and multiple defects. The Journal of Clinical Dysmorphology 1: 5-7.

13. Kittur SD, Weaver DD, Maves MD (1983) Syndrome identification case report 95: congenital fusion of the gums and jaws. Journal of Clinical Dysmorphology 1983: 2-4.

14. Simpson JR, Maves MD (1985) Congenital syngnathia or fusion of the gums and jaws. Otolaryngology - Head And Neck Surgery 93: 96-99.

15. Nwoku AL, Kekere-Ekun TA (1986) Congenital ankylosis of the mandible. Journal of Maxillofacial Surgery 14: 150-152.

16. Brown AG, Marsh JL (1990) Agnathia and associated malformations: a case report. Cleft Palate Journal 27: 415-418.

17. Traaholt LM (1990) Cleft lip and palate associated with alveolar synechia and filiform palbebral synechia. Plastic And Reconstructive Surgery 1990: 337-339.

18. v. Domarus H, Scheunemann H (1990) Congenital prearticular tempo-mandibular ankylosis in two siblings. Journal of Craniofacial and Maxillofacial Surgery 18: 299-303.

19. Gartlan MG, Davies J, Smith RJH (1993) Congenital oral synechiae. Annals Of Otology, Rhinology And Laryngology 102: 186-197.

20. Agrawal K, Chandra SS, Sreekumar NS (1993) Congenital bilateral intermaxillary bony fusion. Annals Of Plastic Surgery 30: 163-166.

21. Arshad AR, Goh CS (1994) Hypoglossia congenita with anterior maxillo-mandibular fusion. British Journal Of Plastic Surgery 47: 139-141.

22. Kamata S, Satoh K, Uemara T, Onizuka T (1996) Congenital bilateral zygomatico-mandibular fusion with mandibular hypoplasia. British Journal Of Plastic Surgery 49: 251-253.

23. Behnia H, Shamse MG (1996) Congenital unilateral fusion of the mandibular and maxillary alveolar ridges, temporomandibular joint, and coronoid process. A case report. Journal Of Oral And Maxillofacial Surgery 54: 773-776.

24. Jackson IT, Agrawal K, Bush K (1996) Congenital bilateral maxillo-mandibulo zygomatic fusion with bilateral oblique facial clefts. European journal of plastic surgery 19: 262-264.

25. Dawson KH, Gruss JS, Myall RW (1997) Congenital bony syngnathia: a proposed classification. Cleft Palate Craniofac J 34: 141-146.

26. Rao S, Oak S, Wagh M, Kulkarni B (1997) Congenital midline palatomandibular bony fusion with a mandibular cleft and a bifid tongue. Br J Plast Surg 50: 139-141.

27. Ugurlu K, Turan T, Urganci N, Gozu A, Gunay Y, et al. (1999) Fusion of maxillary and mandibular alveolar process together with a median mandibular cleft: a rare congenital anomaly. J Craniomaxillofac Surg 27: 105-108.

28. Yazdi I, Fakhraee AH (2000) Congenital fusion of maxilla and mandible (bony syngnathia): a case report. Archives of Iranian Medicine 3.

29. Knoll B, Karas D, Persing JA, Shin J (2000) Complete congenital bony syngnathia in a case of oromandibular limb hypogenesis syndrome. J Craniofac Surg 11: 398-404.

30. Laster Z, Temkin D, Zarfin Y, Kushnir A (2001) Complete bony fusion of the mandible to the zygomatic complex and maxillary tuberosity: case report and review. Int J Oral Maxillofac Surg 30: 75-79.

31. Verloes A, Raoul M, Genevieve D, Sznajer Y, Demarche M, et al. (2004) Bony syngnathia, vertebral segmentation defect, coloboma, microcephaly and mental retardation: confirmation of Dobrow syndrome and review of syndromal syngnathias. Clin Dysmorphol 13: 205-211.

32. Daniels JS (2004) Congenital maxillomandibular fusion: a case report and review of the literature. J Craniomaxillofac Surg 32: 135-139.

33. Ugurlu K, Karsidag S, Huthut I, Yildiz K, Bas L (2005) Congenital fusion of the maxilla and mandible. J Craniofac Surg 16: 287-291.

34. Shams MG, Motamedi MH, Abad HL (2006) Congenital fusion of the maxilla and mandible: brief case report. Oral Surg Oral Med Oral Pathol Oral Radiol Endod 102: e1-3.

35. Nikolic S, Lukac B, Bokun J, Jasovic A (2006) Unilateral mandibulomaxillar synostosis as a single anomaly in a newborn. J Oral Maxillofac Surg 64: 843-846.

36. Mir MA, Hafeez A, Zargar HR, Rasool A, Mohsin M, et al. (2007) Syngnathia without any other associated anomaly: a very rare case report. The Internet Journal of Plastic Surgery 4.

37. Trigg DJ, Mau IT, Rosbe KW (2007) Complete bony syngnathia: Report of a case and review. Arch Otolaryngol Head Neck Surg 133: 187-190.

38. Vahidi K, Joe BN, Glenn OA, Barkovich AJ, Filly R, et al. (2007) Prenatal imaging of congenital maxillomandibular fusion: case report and review of the literature. J Ultrasound Med 26: 381-389.

39. Gupta RK, Jadhav V, Gupta A, Sanghvi B, Shah H, et al. (2008) Congenital alveolar fusion. J Pediatr Surg 43: 1421-1425.

40. Konas E, Tuncbilek G, Kayikcioglu A, Ozgur F (2010) Congenital bony syngnathia with unilateral palatal shelf and soft palate agenesis. J Craniofac Surg 21: 1645-1647.

41. El-Hakim IE, Al-Sebaei MO, Abuzennada S, AlYamani AO (2010) Congenital fusion of the maxilla and mandible (congenital bony syngnathia). Int J Oral Maxillofac Surg 39: 933-936.

42. Fallahi HR, Naeini M, Mahmoudi M, Javaherforoosh F (2010) Congenital zygomatico-maxillo-mandibular fusion: a brief case report and review of literature. Int J Oral Maxillofac Surg 39: 930-933.

43. Halli R, Kharkar V, Kini Y, Rudagi BM (2010) Congenital unilateral maxillo-mandibulo-zygomatic fusion (syngnathia): a case report in an 8-year-old boy. Int J Oral Maxillofac Surg 39: 500-502.

44. Naikmasur VG, Sattur AP, Joshi SK, Rai A (2010) Congenital syngnathia: case report and review of literature. Cleft Palate Craniofac J 47: 654-660.

45. Subramanian B, Agrawal K, Panda K (2010) Congenital fusion of the jaws: a management protocol. Int J Oral Maxillofac Surg 39: 925-929.

46. Tauro DP, Kalappanavar NK, Kiran HY, Girhe VJ (2011) Congenital zygomatico-mandibular fusion (pseudo-syngnathia?) in conjunction with unilateral anophthalmia: Review of terminology & classification. Cleft Palate Craniofac J.
