# Supplementary material for: Interaction between Foxc1 and Fgf8 during Mammalian Jaw Patterning and in the Pathogenesis of Syngnathia
Source: PLoS Genet. 2013 Dec 19;9(12):e1003949. doi: 10.1371/journal.pgen.1003949 (PMC3868537; doi:10.1371/journal.pgen.1003949)
Supplement: Table S2 — Genotyping primer sequences. Summary of the sequences used for genotyping and RT-PCR. (DOC) [file pgen.1003949.s008.doc]

**Table S2.** Genotyping primer sequences

| Probe Name | Forward Primer | Reverse Primer | Reporter |
| --- | --- | --- | --- |
| Foxc1-WT | GCCTGGACGCTGCAGAA | CATGATGTTGTCCACGCTGAAG | CCGCCGCATCACAG |
| Foxc1-KO | CCTACAGCTACATCGCTCTTATCAC | TCTCTTCTTTTTTGGAGCCATGGT | CATGGTACCACGTGGTTTA |
| Fgf8-1 WT | CCAACCCATCCGCAAATGAG | TCCTATCCCTGGTACAGTTGCT | CTGCAGCCTGTGCTTT |
| Fgf8-1-EX | GGCCGAAGTTCCTATTCTCTAGAAA | GATTCCTCGGCCATAACTTCGTAT | CGAATAACTTCGTATAGCATACATT |
| Fgf8-Neo | GGGCGCCCGGTTCTT | CCTCGTCCTGCAGTTCATTCA | ACCTGTCCGGTGCCC |
| Cre | TTAATCCATATTGGCAGAACGAAAACG | CAGGCTAAGTGCCTTCTCTACA | CCTGCGGTGCTAACC |
| Z/EG-1 TG | CGGTATCGATAAGCAGCTTGATGA | CCCAGTCACGACGTTGTAAAAC | ACATGGCGGATCCCGTC |
